# Pharmacological difference between degrader and inhibitor against oncogenic BCR-ABL kinase

**DOI:** 10.1038/s41598-018-31913-5

**Published:** 2018-09-10

**Authors:** Norihito Shibata, Kenichiro Shimokawa, Katsunori Nagai, Nobumichi Ohoka, Takayuki Hattori, Naoki Miyamoto, Osamu Ujikawa, Tomoya Sameshima, Hiroshi Nara, Nobuo Cho, Mikihiko Naito

**Affiliations:** 1 Divisions of Molecular Target and Gene Therapy Products, National Institute of Health Sciences, 3-25-26 Tonomachi, Kawasaki-ku, Kawasaki, Kanagawa 210-9501 Japan; 2 Pharmaceutical Research Division, Takeda Pharmaceutical Co. Ltd., 2-26-1 Muraoka-Higashi, Fujisawa, Kanagawa, 251-8555 Japan; 3 Present Address: Axcelead Drug Discovery Partners, Inc., 2-26-1 Muraoka-Higashi, Fujisawa, Kanagawa, 251-0012 Japan; 4 Present Address: The Pharmaceutical Society of Japan, 2-12-15 Shibuya, Shibuya-ku, Tokyo, 150-0002, Japan; 5 Present Address: Drug Discovery Chemistry Platform Unit (Wako branch), RIKEN Center for Life Science Technologies, 2-1 Hirosawa, Wako, Saitama, 351-0198, Japan

## Abstract

Chronic myelogenous leukemia (CML) is characterized by the oncogenic fusion protein, BCR-ABL protein kinase, against which clinically useful inhibitors have been developed. An alternative approach to treat CML is to degrade the BCR-ABL protein. Recently, potent degraders against BCR-ABL have been developed by conjugating dasatinib to ligands for E3 ubiquitin ligases. Since the degraders contain the dasatinib moiety, they also inhibit BCR-ABL kinase activity, which complicates our understanding of the impact of BCR-ABL degradation by degraders in CML growth inhibition. To address this issue, we chose DAS-IAP, as a potent BCR-ABL degrader, and developed a structurally related inactive degrader, DAS-meIAP, which inhibits kinase activity but does not degrade the BCR-ABL protein. DAS-IAP showed slightly weaker activity than DAS-meIAP in inhibiting cell growth when CML cells were treated for 48 h. However, DAS-IAP showed sustained growth inhibition even when the drug was removed after short-term treatment, whereas CML cell growth rapidly resumed following removal of DAS-meIAP and dasatinib. Consistently, suppression of BCR-ABL levels and downstream kinase signaling were maintained after DAS-IAP removal, whereas kinase signaling rapidly recovered following removal of DAS-meIAP and dasatinib. These results indicate that BCR-ABL degrader shows more sustained inhibition of CML cell growth than ABL kinase inhibitor.

## Introduction

Chronic myelogenous leukemia (CML) is a myeloproliferative disorder characterized by the fusion gene *Bcr-Abl*, which is generated by a chromosomal translocation of the *Abl* gene on chromosome 9 to the *Bcr* gene on chromosome 22 to give a constitutively active protein tyrosine kinase, BCR-ABL^1–5^. The kinase activity of BCR-ABL activates downstream signaling and thus causes unregulated proliferation of CML cells in patients. Several BCR-ABL tyrosine kinase inhibitors (TKIs) have been discovered and approved for CML treatment^6–9^. These TKIs are capable of saving most CML patients; however, a significant number of patients develop drug resistance, which is commonly caused by point mutations in the tyrosine kinase domain of BCR-ABL. Therefore, novel kinase inhibitors are constantly being developed in an effort to overcome drug resistance.

An alternative to the inhibition of BCR-ABL kinase activity is the downregulation of BCR-ABL protein, which should have a potential therapeutic effect. Recently, we and others have developed protein knockdown technologies, which induces the degradation of target proteins using hybrid small molecules named SNIPERs (Specific and Non-genetic inhibitor of apoptosis protein [IAP]-dependent Protein Erasers)^10–25^ and PROTACs, (Proteolysis Targeting Chimeras)^26–43^. SNIPERs and PROTACs are chimeric molecules composed of two different ligands connected by a linker; one ligand is for the target protein and the other is for E3 ubiquitin ligases. Accordingly, these molecules are expected to crosslink the target protein and E3 ubiquitin ligases in cells, resulting in the ubiquitylation and subsequent degradation of the target protein via the ubiquitin-proteasome system (UPS). Currently, several oncogenic proteins, *e.g*., androgen receptor^23,36,37^, estrogen receptor^17,19,35^, transforming acidic coiled-coil containing protein 3^15,16^, c-Met^43^ and bromodomain containing 4^28,29,31,33,39–42^, have been degraded effectively via the UPS using the protein knockdown technology. Recently, degraders of BCR-ABL have also been developed^11,22,24,30^.

To induce target protein degradation, the degraders require a ligand moiety that has binding affinity to a target protein, which is dasatinib in potent degraders against BCR-ABL. The BCR-ABL degraders were found to reduce effectively the BCR-ABL protein level and to suppress the growth of BCR-ABL-positive CML cells^22,30^. However, the degraders also inhibit BCR-ABL tyrosine kinase activity, which complicates the value and suitability of BCR-ABL degradation in CML cell growth inhibition by degraders.

In this study, we compared directly the activity of BCR-ABL degraders recruiting different E3 ubiquitin ligases. We also compared the inhibitory activity of the degrader and kinase inhibitors against CML cell growth. We found that drug removal after short-term treatment with inhibitors resulted in the rapid recovery of CML cell growth, whereas use of a degrader showed more sustained CML growth inhibition under the same condition. We discuss a possible mechanism of the sustained inhibition of CML cell growth by BCR-ABL degraders.

## Results

### Comparison of various BCR-ABL degraders recruiting different E3 ubiquitin ligases

**Figure 1:**
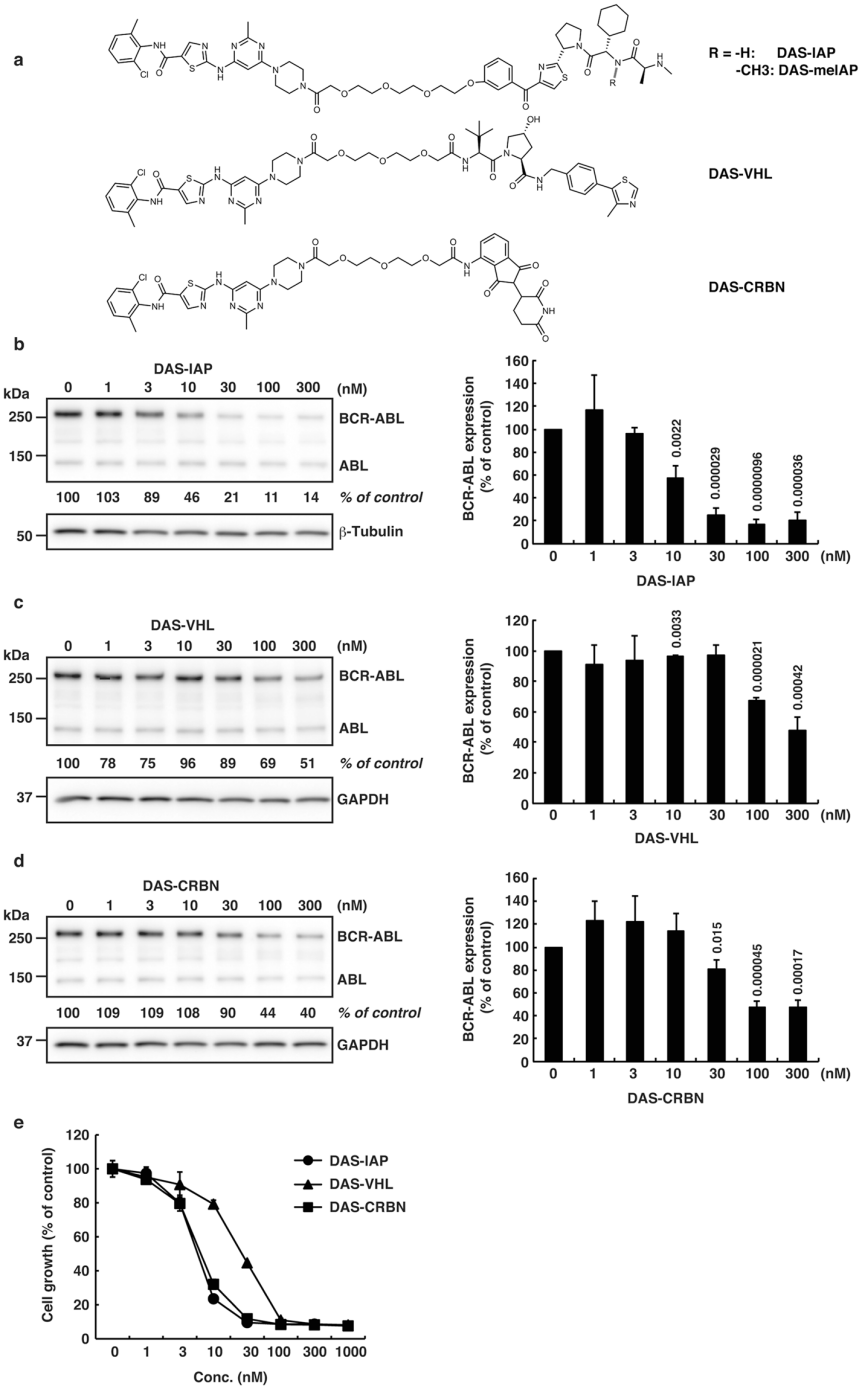
Protein knockdown and growth inhibition by the designed conjugates composed of dasatinib and ligands for E3 ligases (IAP, VHL or CRBN). (**a**) Chemical structures of the designed conjugates. (**b**–**d**) K562 cells were incubated with the indicated concentration of the conjugate for 6 h. Numbers below the BCR-ABL panel represent BCR-ABL/β-tubulin or BCR-ABL/GAPDH ratios normalized by the vehicle control as 100. Data in the bar graph are means ± SD (*n* = 3). *P* < 0.05 compared with the vehicle control are considered to be significant and actual *P* values are presented. In Fig. 1c, the reduction of BCR-ABL protein by DAS-VHL at 10 nM happened to be statistically significant. However, we think it is not pharmacologically significant, because the reduction is very little and DAS-VHL at 1, 3, and 30 nM did not show statistically significant effect on the protein level of BCR-ABL. (**e**) Cells were incubated with the indicated concentration of the conjugate for 48 h and subjected to the WST assay. Data in the graph are means ± SD (*n* = 3).

**Figure 2:**
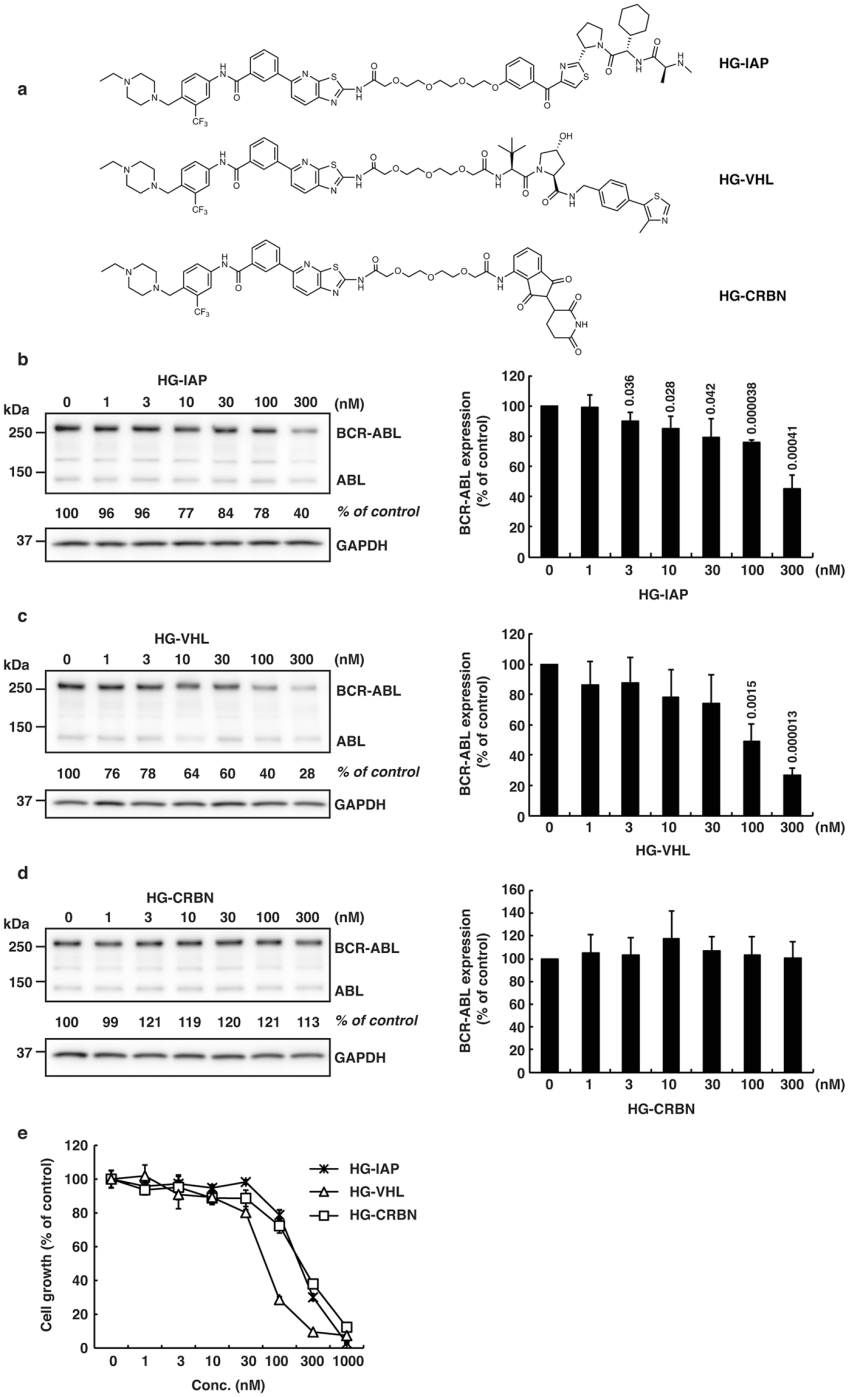
Protein knockdown and growth inhibition by the designed conjugates composed of HG-7-85-01 and ligands for E3 ligases (IAP, VHL or CRBN). (**a**) Chemical structures of the designed conjugates. (**b**–**d**) K562 cells were incubated with the indicated concentration of the conjugate for 6 h. Numbers below the BCR-ABL panel represent BCR-ABL/GAPDH ratios normalized by the vehicle control as 100. Data in the bar graph are means ± SD (*n* = 3). *P* < 0.05 compared with the vehicle control are considered to be significant and actual *P* values are presented. (**e**) Cells were incubated with the indicated concentration of the conjugate for 48 h and subjected to the WST assay. Data in the graph are means ± SD (*n* = 3).

Recently, we and other groups have developed degraders against the oncogenic BCR-ABL protein^11,22,24,30^. These degraders were designed to recruit various E3 ubiquitin ligases (IAP, von Hippel-Lindau (VHL) or Cereblon (CRBN)) by containing different E3 ligands. To compare directly the protein knockdown activity of the degraders against BCR-ABL, we have conjugated ABL inhibitors (dasatinib^44^ or HG-7-85-01^45^) to ligands for E3 ligases (an LCL-161 derivative to recruit IAPs^46^, a known VHL ligand^47^, or pomalidomide to recruit CRBN^48^) with a polyethylene glycol × 3 linker (Figs 1a, 2a). The resulting hybrid molecules were assayed for binding affinity to ABL1, as determined by a time-resolved fluorescence resonance energy transfer (TR-FRET)-based binding assay and showed potent binding to ABL1 with affinities of 10^−10^ to 10^−7^ M (data not shown).

These degraders were then examined for their activity to degrade BCR-ABL in K562 cells for 6 h (Figs 1b–d and 2b–d) and to suppress the growth of K562 cells for 48 h (Figs 1e, 2e). When dasatinib was incorporated as a target ligand, IAP- and CRBN-based degraders (DAS-IAP and DAS-CRBN) showed effective knockdown activity against the BCR-ABL protein, whereas the VHL-based degrader (DAS-VHL) showed negligible activity (Fig. 1b–d). Consistent with the downregulation of the BCR-ABL protein, DAS-IAP and DAS-CRBN showed more potent suppression of K562 cell growth than DAS-VHL (Fig. 1e). However, when HG-7-85-01 (another inhibitor of BCR-ABL) was incorporated, the VHL-based degrader (HG-VHL) showed effective protein knockdown activity, whereas IAP-based (HG-IAP) and CRBN-based (HG-CRBN) degraders showed weak and no activity, respectively (Fig. 2b–d). Consistent with the downregulation of the BCR-ABL protein, HG-VHL showed more potent suppression of the growth of K562 cells than HG-IAP and HG-CRBN (Fig. 2e). These results suggest that DAS-IAP, DAS-CRBN, and HG-VHL can recruit E3 ligases to a suitable position so that the lysine residues on the surface of BCR-ABL can be ubiquitylated. Thus, the appropriate combination of an E3 ligand and target ligand is critically important for protein knockdown activity of the degraders. Since DAS-IAP, composed of dasatinib and an IAP ligand LCL161 derivative, showed the most potent degradation activity, we chose DAS-IAP for further study.

### DAS-IAP shows slightly weaker activity than DAS-meIAP to inhibit cell growth

**Figure 3:**
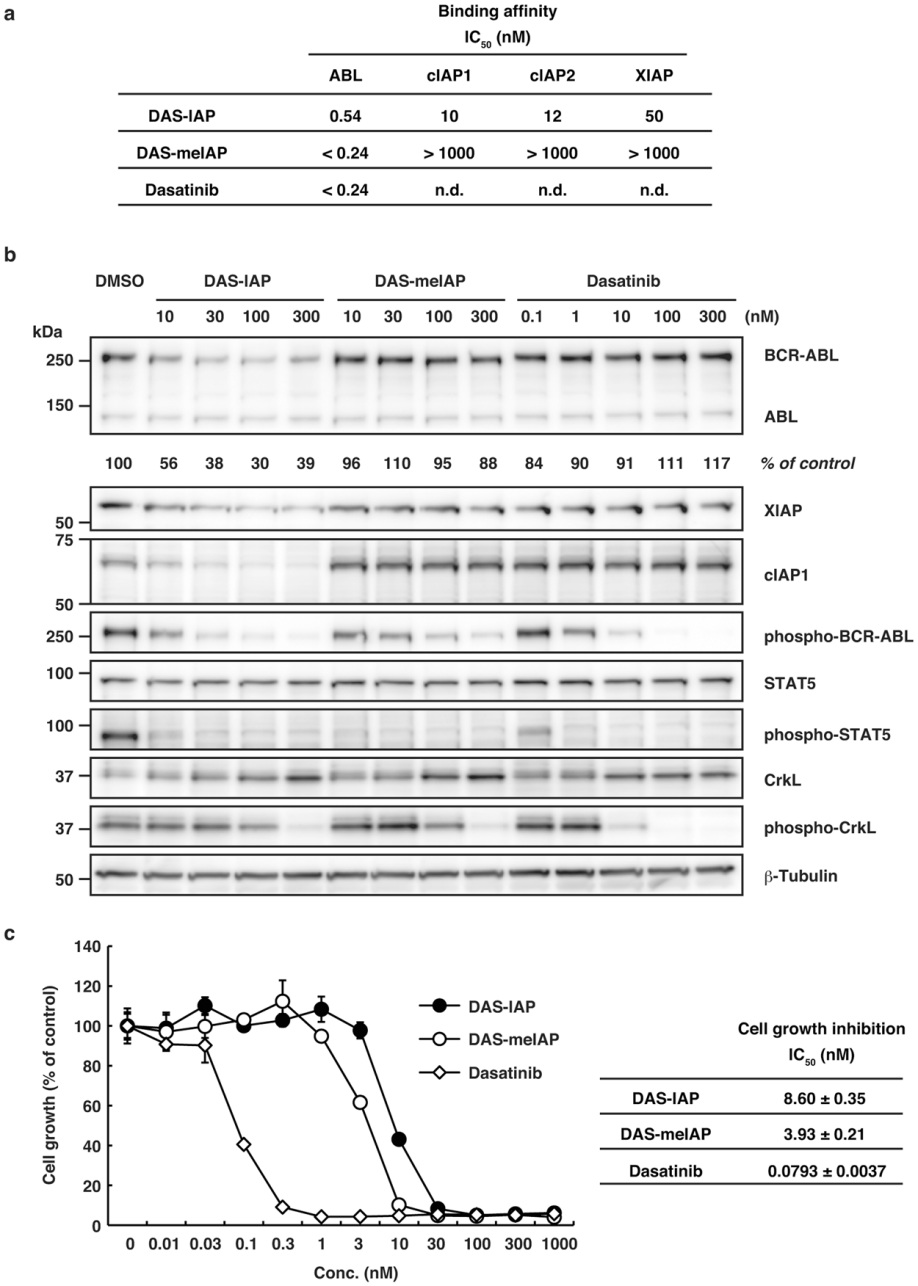
Binding affinities, protein knockdown activities and growth inhibitory effects of DAS-IAP, DAS-meIAP and dasatinib. (**a**) Binding affinities of the degraders and dasatinib to ABL and IAP. IC_50_ values (concentration of the compounds required to inhibit the probe binding to ABL by 50%) are presented. n.d.: not determined. (**b**) K562 cells were incubated with the indicated concentration of the drugs for 6 h. Numbers below the BCR-ABL panel represent BCR-ABL/β-tubulin ratios normalized by the vehicle control as 100. (**c**) K562 cells were incubated with the indicated concentration of the drugs for 48 h and subjected to the WST assay. IC_50_ values are presented as means ± SD (*n* = 3).

As we previously reported^22^, DAS-IAP is a potent BCR-ABL protein degrader that is dependent on the ubiquitin system and IAPs. In addition to the degradation, DAS-IAP also inhibits ABL kinase activity, because it contains the dasatinib moiety as a target ligand that inhibits ABL kinase activity. To investigate the importance of BCR-ABL degradation in CML growth inhibition by DAS-IAP, we developed a structurally related inactive degrader as a control compound, which is composed of dasatinib and an N-methylated LCL161 derivative (DAS-meIAP) that cannot recruit IAPs. Figure 3a summarizes the binding affinity of DAS-IAP, DAS-meIAP and dasatinib to ABL1, cIAP1, cIAP2 and XIAP. DAS-meIAP showed strong affinity towards ABL1, but lost the ability to bind IAP proteins (Fig. 3a).

In CML, such as K562 cells, the constitutively active tyrosine kinase BCR-ABL promotes unregulated cell proliferation through phosphorylation signaling pathways that involve signal transducer and activator of transcription 5 (STAT5) and Crk-like proto-oncogene (CrkL). Therefore, we examined the activity of DAS-IAP and DAS-meIAP to degrade the BCR-ABL protein and to suppress downstream signaling pathways in K562 cells for 6 h. DAS-IAP reduced BCR-ABL, cIAP1 and XIAP protein levels and suppressed phosphorylation of BCR-ABL, STAT5 and CrkL (Fig. 3b), as previously reported^22^. DAS-meIAP and dasatinib also suppressed phosphorylation of BCR-ABL and the downstream substrates STAT5 and CrkL in accordance with the binding affinity toward ABL; however, DAS-meIAP and dasatinib did not reduce BCR-ABL, cIAP1 and XIAP protein levels (Fig. 3b), indicating that DAS-meIAP and dasatinib inhibits kinase activity but does not degrade BCR-ABL.

We then examined the activity of DAS-meIAP to suppress the growth of K562 cells for 48 h. In line with the inhibition of the BCR-ABL kinase signaling pathways, DAS-meIAP inhibited the growth of K562 cells with an IC_50_ value of 3.93 nM, which was slightly higher in activity than DAS-IAP with an IC_50_ value of 8.60 nM (Fig. 3c). Dasatinib was 100 times more active than DAS-IAP to inhibit CML cell growth under this condition. These results suggest that although DAS-IAP degrades the BCR-ABL protein, the inhibition of BCR-ABL kinase activity is more apparently involved in the inhibition of kinase signaling and CML cell growth under continuous exposure to the drug. We also examined the effect of these drugs on the growth of MOLT-4 and Jurkat cells, which do not express BCR-ABL protein. In accordance with our previous report^22^, these drugs showed IC_50_ values of >1 µM in MOLT-4 and Jurkat cells (Supplementary Fig. S1), which were 200 times higher than those in K562 cells. These data suggest that the inhibitory effect of these drugs is highly selective to CML cells expressing BCR-ABL protein.

### Sustained growth inhibition by DAS-IAP after drug removal

**Figure 4:**
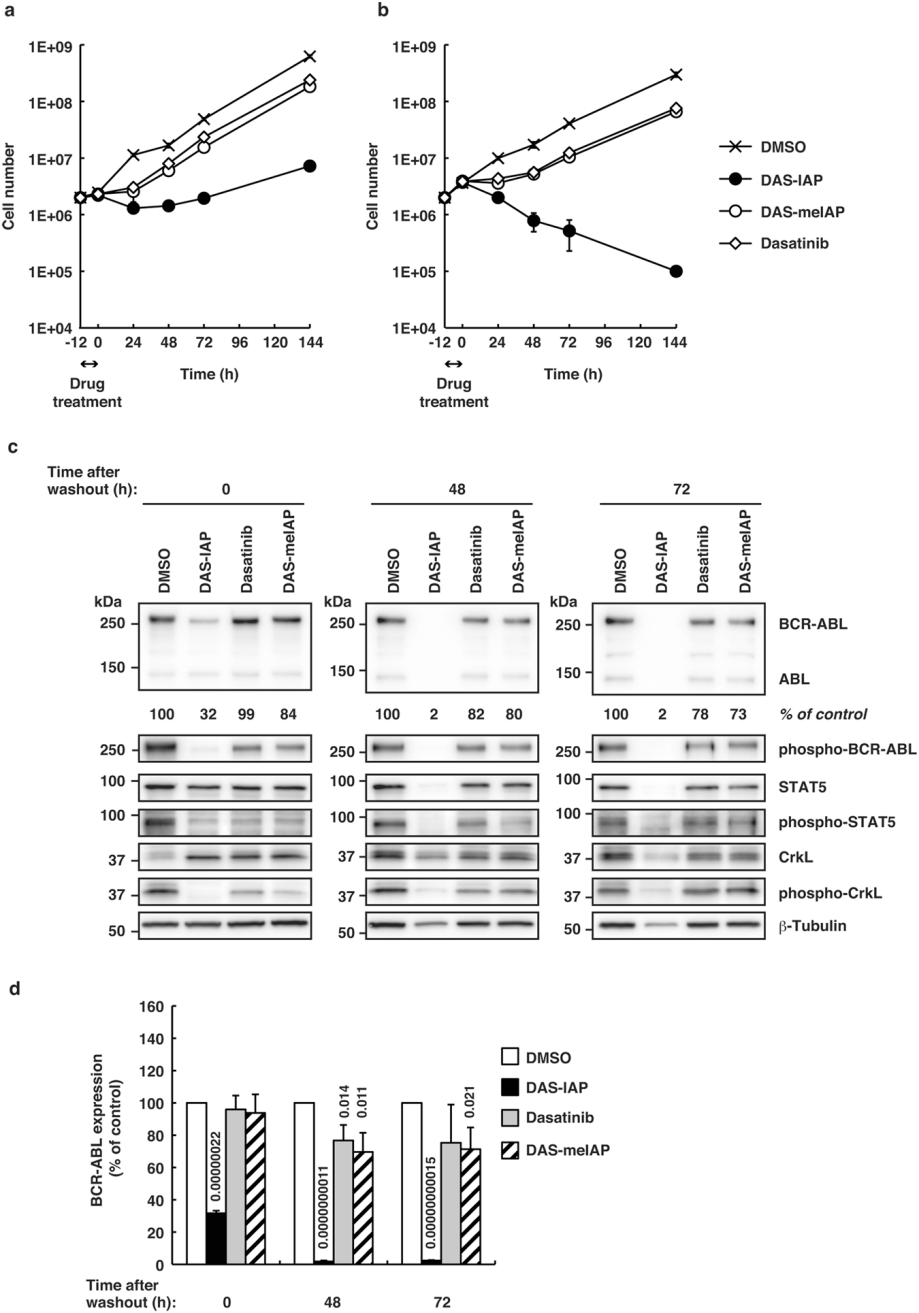
Sustained growth inhibition, and suppression of BCR-ABL protein and downstream kinase signaling by DAS-IAP after drug removal in K562 cells. Cells were treated with 25 (**a**) or 50 times (**b**) higher concentration than the IC_50_ of each drug for 12 h, washed three times to remove the drugs and incubated in drug-free medium. Cells were counted at the indicated times. Data were presented as means ± SD (*n* = 3). (**c**,**d**) Cells were treated with 50 times higher concentration than the IC_50_ of each drug for 12 h, washed three times to remove the drugs and incubated in drug-free medium. Effect of DAS-IAP, DAS-meIAP, and dasatinib on BCR-ABL protein level and the downstream kinase signaling. Numbers below the BCR-ABL panel represent BCR-ABL/β-tubulin ratios normalized by the vehicle control as 100. Data in the bar graph are means ± SD (*n* = 3). *P* < 0.05 compared with the vehicle control are considered to be significant and actual *P* values are presented.

Clinical pharmacokinetic studies revealed that dasatinib has a short (3 to 5 h) plasma half-life^49^, suggesting that suppression of CML cell growth by dasatinib may be transient in CML patients. Therefore, we examined the effect of short-term treatment with the drugs on the growth of CML cells. K562 cells were treated with 25 or 50 times higher concentrations than the IC_50_ of each drug for 12 h, and then the cells were washed and incubated in drug-free medium for 6 d (Fig. 4a,b). After the pulsed treatment for 12 h, kinase inhibitors (dasatinib and DAS-meIAP) slightly delayed the growth of K562 cells; however, the cells resumed proliferating rapidly after drug removal. In contrast, DAS-IAP markedly inhibited the growth of K562 cells even after drug removal. The K562 cell number decreased continuously at a DAS-IAP concentration 50 times higher than the IC_50_, which contrasts strikingly to the kinase inhibitors tested (Fig. 4b).

**Figure 5:**
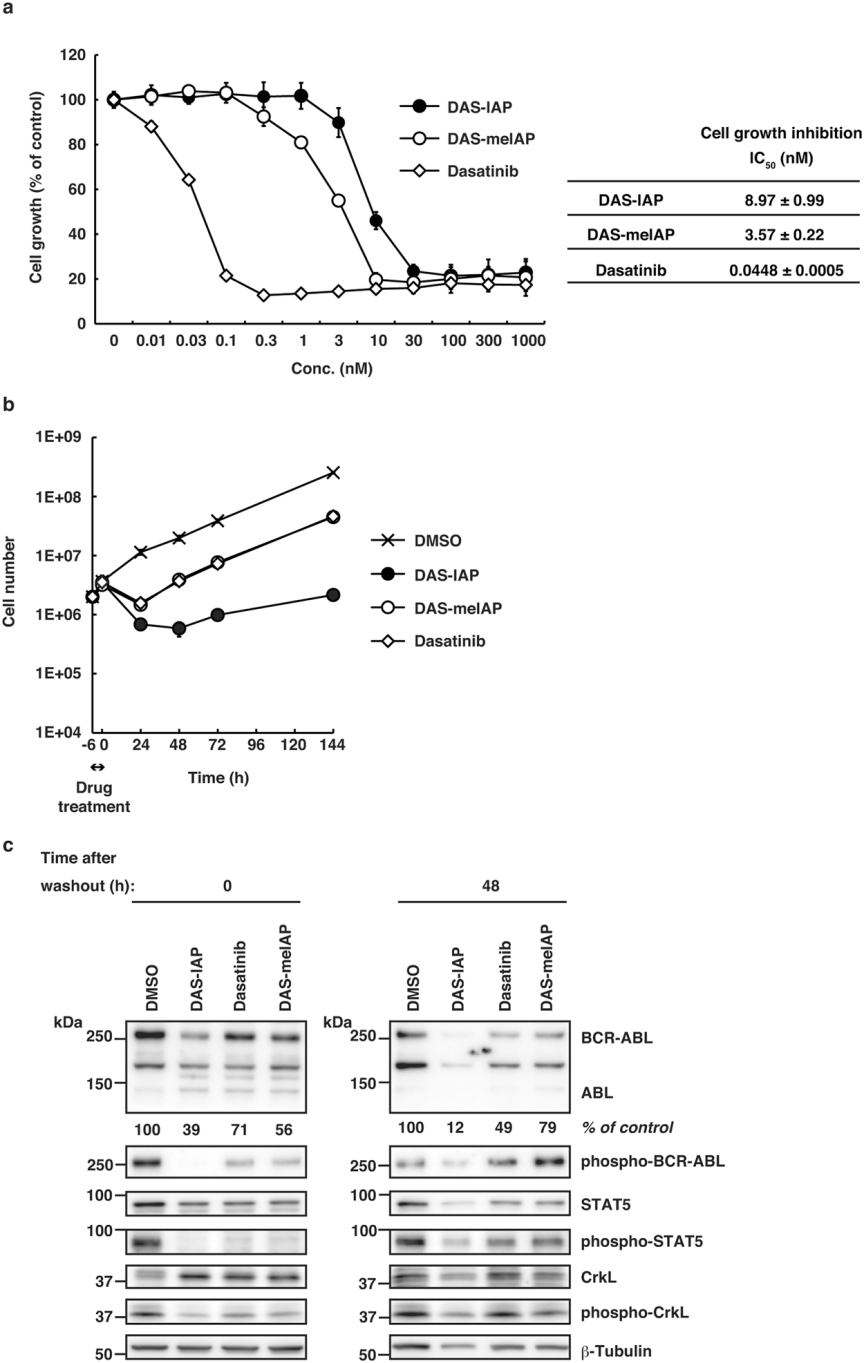
Sustained growth inhibition, and suppression of BCR-ABL protein and downstream kinase signaling by DAS-IAP after drug removal in KU812 cells. (**a**) Cells were incubated with the indicated concentration of the drugs for 48 h and subjected to the WST assay. IC_50_ values are presented as means ± SD (*n* = 3). (**b**,**c**) Cells were treated with 50 times higher concentration than the IC_50_ of each drug for 6 h, washed three times to remove the drugs and incubated in drug-free medium. (**b**) Cells were counted at the indicated times. Data were presented as means ± SD (*n* = 3). (**c**) The cell lysates were analyzed by Western blot. Numbers below the BCR-ABL panel represent BCR-ABL/β-tubulin ratios normalized by the vehicle control as 100.

We then examined the BCR-ABL protein level and downstream signaling in K562 cells pulse-treated for 12 h with 50 times higher concentration than the IC_50_ (Fig. 4c,d). Treating the cells with DAS-IAP, DAS-meIAP and dasatinib for 12 h (time 0) inhibited the phosphorylation of BCR-ABL, STAT5 and CrkL, indicating that kinase signaling was effectively inhibited by these drugs. The phosphorylation of BCR-ABL was more prominently diminished in the DAS-IAP-treated cells than in cells treated with DAS-meIAP and dasatinib, probably because the BCR-ABL protein level is seriously reduced in the DAS-IAP-treated cells. At 48 and 72 h after the drug removal, the phosphorylated BCR-ABL, STAT5 and CrkL recovered in cells treated with the kinase inhibitors, whereas they remained significantly diminished along with the BCR-ABL protein levels in the DAS-IAP-treated cells. At 144 h after the drug removal, cells were destroyed almost completely and we could not obtain enough amount of protein sample for western blot analysis with the DAS-IAP-treated cells (data not shown). Similar results were observed in another CML cell line, KU812, expressing dasatinib-sensitive BCR-ABL protein (Fig. 5). These results strongly suggest that CML cell growth suppression by short-term treatment with DAS-IAP is due to degradation of the BCR-ABL protein and not to ABL kinase inhibition, implying that cell growth inhibition by degradation of BCR-ABL is sustained longer than that by inhibition of BCR-ABL kinase activity.

**Figure 6:**
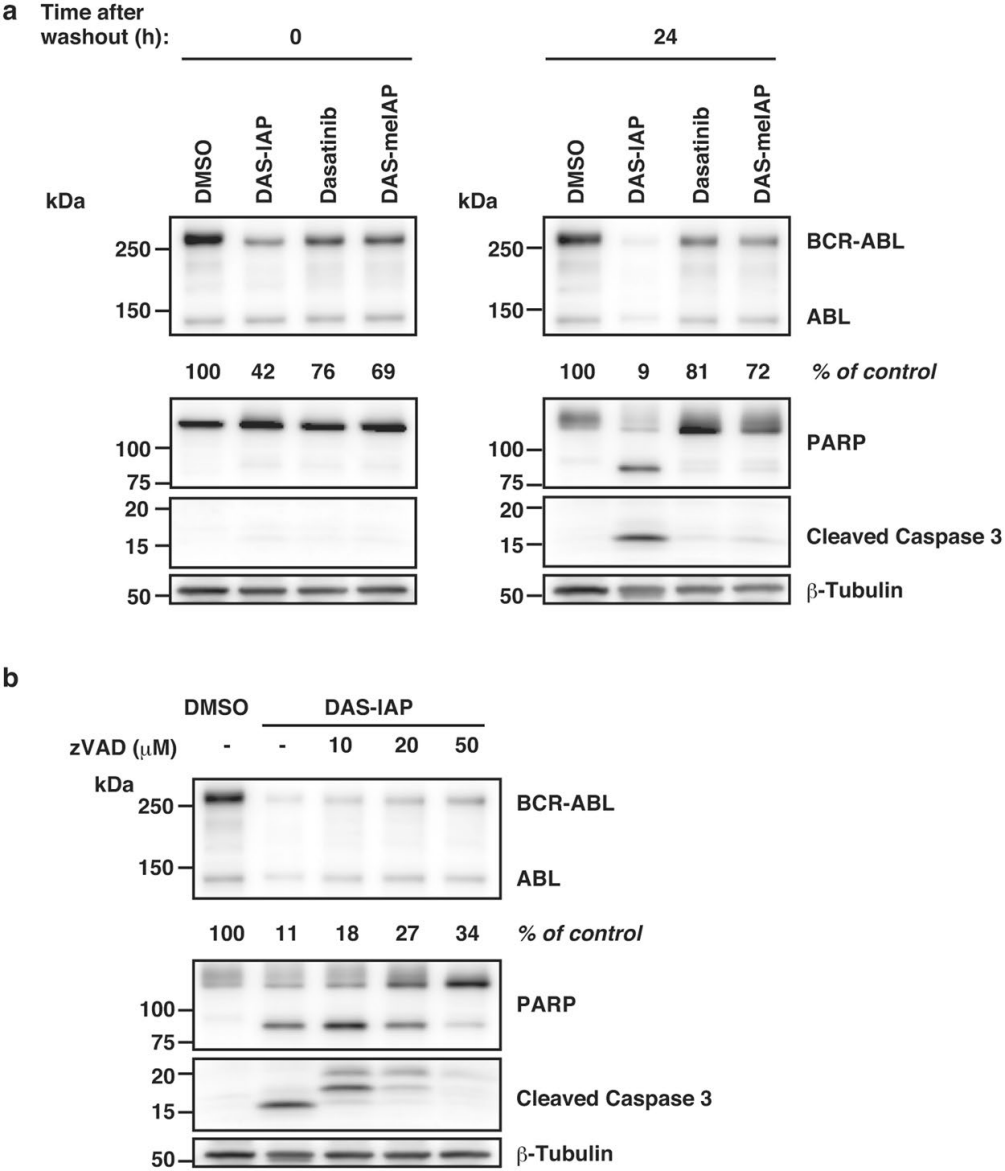
Effect of DAS-IAP on caspase activation after drug removal in K562 cells. (**a**,**b**) Cells were treated with 50 times higher concentration than the IC_50_ of each drug for 12 h, washed three times to remove the drugs and incubated in drug-free medium. After drug removal, cells were incubated with or without the indicated concentrations of zVAD-FMK for 24 h. Numbers below the BCR-ABL panel represent BCR-ABL/β-tubulin ratios normalized by the vehicle control as 100.

Finally, we examined whether the degradation of BCR-ABL protein by DAS-IAP induces apoptosis. K562 cells were pulse-treated for 12 h with 50 times higher concentration of IC_50_. Drug removal for 24 h showed that caspase 3 activation and poly (ADP-ribose) polymerase (PARP) cleavage were induced in cells treated with DAS-IAP, but not in cells treated with DAS-meIAP and dasatinib (Fig. 6a). The caspase activation and PARP cleavage by DAS-IAP were abrogated by a pan-caspase inhibitor, zVAD-FMK (Fig. 6b). These results suggest that the degradation of BCR-ABL protein by DAS-IAP results in the growth inhibition and the apoptosis induction in CML cells.

## Discussion

Protein degradation by SNIPERs and PROTACs has attracted considerable attention as a platform technology for novel drug discovery. Currently, several degraders against oncogenic proteins have been developed successfully, and some of these degraders suppress the growth of tumor cells *in vivo*^17,33,39,42^. Therefore, this approach provides a highly promising new modality for drug discovery. However, a clear link between degradation of the target protein by the degraders for ensuring suppression of tumor growth remains unresolved because many degraders contain an inhibitor moiety as a ligand for the target protein. In this study, we selected DAS-IAP, as a potent BCR-ABL degrader, and developed a structurally related inactive degrader, DAS-meIAP, which inhibits kinase activity but does not degrade BCR-ABL. We compared the effect of DAS-IAP and DAS-meIAP on CML cell growth, and found that growth suppression by short-term treatment with DAS-IAP is due to degradation of BCR-ABL and not to the inhibition of BCR-ABL kinase activity. Moreover, growth suppression by degradation of BCR-ABL is sustained longer than that by inhibition of BCR-ABL kinase activity. To confirm the feature of the degraders, it is insightful to develop a molecule that would not inhibit the kinase, but would only degrade BCR-ABL protein. BCR-ABL protein has multiple domains such as PH, SH2 and SH3 domains, to which novel ligands could be developed. Incorporation of such ligands into degrader would allow us to develop novel degraders that can induce degradation of BCR-ABL protein without inhibition of the kinase activity. Furthermore, these degraders would induce degradation of BCR-ABL protein irrespective of the mutations in kinase domain that confer resistance against kinase inhibitors.

Short-term treatment with DAS-IAP showed long-lasting suppression of the proliferation of CML cells, whereas DAS-meIAP and dasatinib slightly retard the growth of CML cells after drug removal (Figs 4a,b and 5b). Consistently, short-term treatment with DAS-IAP markedly reduced the level of the BCR-ABL protein, which was maintained thereafter, whereas inhibition of BCR-ABL kinase activity by short-term treatment with DAS-meIAP and dasatinib was transient (Figs 4c,d and 5c). These results raise the question as to why degradation by DAS-IAP is more sustained than the kinase inhibition by DAS-meIAP and dasatinib. A possible explanation is the difference in the event-driven and the occupancy-driven pharmacology model^50,51^. Inhibition of kinase activity is an occupancy-based strategy; kinase inhibitors, such as DAS-meIAP and dasatinib must bind to a target kinase to inhibit their activity. Therefore, high concentrations of the inhibitors should be maintained to ensure active-site occupancy and to sustain the inhibitory effect. In contrast, degradation of oncogenic proteins is an event-driven strategy; degraders, such as DAS-IAP, suppress protein function by reducing the target protein level. Therefore, CML cells could not drive cell proliferation following DAS-IAP treatment until sufficient amounts of BCR-ABL accumulate in the cells by *de novo* synthesis of the protein, which occasionally results in apoptosis of the cells (Fig. 6).

From the clinical perspective, we speculate the sustained suppression of cell growth by degraders might benefit CML patients. Currently, imatinib is a frontline therapy for CML and dasatinib is effective for treating CML patients who fail imatinib therapy^7,44^. These TKIs are clinically effective with once or twice a day dosing. Although some clinical trials, such as the Stop Imatinib trial^52,53^ and JALSG-STIM213 study^54^, suggest that CML patients who maintained deep molecular response for more than 2 years could safely stop imatinib, the Japanese Society of Hematology guidelines and the European LeukemiaNet guidelines recommend that CML patients should continue TKIs treatment throughout their lifetime^54,55^. Our results suggest that BCR-ABL degraders, such as DAS-IAP, might be an effective alternative. In order to clinically develop the BCR-ABL degraders, it is important to examine the protein knockdown and anti-proliferation activities in primary CML cells and CD34+ stem cells, which allows us to estimate the on- and off-target effects. We also need to investigate the ADMET (absorption, distribution, metabolism, excretion, and toxicity) aspects of the BCR-ABL degraders and to optimize the concentration and treatment regimen of BCR-ABL degraders for maximal clinical impact. In addition, the efficacy and the potential benefit of BCR-ABL degraders should be carefully evaluated in clinical trials. Further studies are required to develop clinically useful degraders against BCR-ABL protein.

## Methods

### Design and Synthesis of BCR-ABL Degraders

We designed hybrid molecules, in which an ABL inhibitor is linked to a ligand of various E3 ligases (IAPs, VHL and CRBN) via a polyethylene glycol linker. DAS-IAP and HG-IAP (“SNIPER(ABL)-39” and “SNIPER(ABL)-33” in reference^22^, respectively) were synthesized as described previously^22^. The chemical synthesis and physicochemical data on the other degraders are provided in the Supplemental Information Doc. S1 and Scheme S1–S3.

### Reagents

Tissue culture plastics were purchased from Greiner Bio-One (Frickenhausen, Germany). The biotinylated anti-His antibody (PV6090) and the His-tagged ABL1 protein (full length, P3049) were from Life Technologies (Carlsbad, CA, USA). Brij^(R)^ 35 solution was obtained from Merck Millipore (Billerica, MA, USA). Terbium-labeled streptavidin (Tb-SA) was from Cisbio (Codolet, France). The recombinant His-tagged human XIAP (BIR3, Asn252-Thr356, 895-XB-050) protein was purchased from R&D Systems (Minneapolis, MN, USA). Recombinant His-tagged human cIAP1 (BIR3, Leu250-Gly350) and cIAP2 (BIR3, Gln238-Ser349) proteins were expressed in *E. coli* and purified using a Ni-NTA column and gel filtration chromatography. The FITC-labeled Smac peptide (FITC-Smac, AVPIAQK(5-FAM)-NH2)^56^ was synthesized in Scrum (Tokyo, Japan). The BODIPY-FL labeled dasatinib (BODIPY-dasatinib)^57^ was synthesized as described previously. zVAD-FMK was purchased from Peptide Institute (Osaka, Japan).

### Cell Culture

Human CML (K562 and KU812 cells), acute T-lymphoblast leukemia (MOLT-4 cell), and T cell leukemia (Jurkat cell) were cultured in Roswell Park Memorial Institute-1640 medium (Sigma-Aldrich, St. Louis, MO, USA) containing 10% fetal bovine serum (Thermo Fisher Scientific, Waltham, MA, USA) and 50 μg/ml kanamycin (Sigma-Aldrich). KU812 cells were obtained from Japanese Collection of Research Bioresources (JCRB, Osaka, Japan) Cell Bank (JCRB0104)^58^.

### Western Blot Analysis

Cells were collected and lysed in a lysis buffer (0.5% Triton X-100, 0.01 M Tris-HCl (pH 7.5), 0.15 M NaCl, Complete Mini protease inhibitor cocktail (Roche Applied Science, Indianapolis, IL, USA) and PhosStop phosphatase inhibitor cocktail (Roche Applied Science)). Protein concentrations were measured by the BCA method (Thermo Fisher Scientific) and an equal amount of protein lysate was separated by SDS-PAGE, transferred to polyvinylidene difluoride membranes (Merck Millipore) and analyzed by western blot using an appropriate antibody. The immunoreactive proteins were visualized using Clarity Western ECL substrate (Bio-Rad, Hercules, CA, USA) and their light emission was quantified with a LAS-3000 lumino-image analyzer (Fuji, Tokyo, Japan). The following antibodies were used: anti-cAbl rabbit polyclonal antibody (pAb) (#2862), anti-XIAP rabbit pAb (#2042), anti-phospho-cAbl rabbit pAb (#3009), anti-STAT5 rabbit pAb (#9363), anti-phospho-STAT5 rabbit pAb (#9359), anti-CrkL mouse monoclonal antibody (mAb) (#3182) and anti-phospho-CrkL rabbit pAb (#3181), anti-PARP rabbit pAb (#9532), and anti-cleaved Caspase 3 rabbit pAb (#9664) (Cell Signaling Technology, Danvers, MA, USA); anti-β-tubulin (ab6046) rabbit pAb (Abcam, Cambridge, UK) and anti-GAPDH rabbit pAb (sc-25778 HRP) (Santa Cruz, Dallas, TX, USA); anti-cIAP1 goat pAb (AF8181) (R&D systems).

### Time-Resolved FRET (TR-FRET) Assay and Data Analysis

The TR-FRET assay was carried out using 384-well white flat-bottom plates (Greiner Bio-One) and the signal was measured using an EnVision Multilabel Plate Reader (PerkinElmer, Waltham, MA, USA). The solution in each well was excited with a laser (λ = 337 nm) reflected by a dichroic mirror (D400/D505 (Perkin Elmer) and fluorescence from terbium (Tb) and BODIPY or FITC was detected through two emission filters (CFP 486 (Perkin Elmer) for Tb and Emission 515 (Perkin Elmer) for BODIPY and FITC). The assay buffer used in this study was composed of 50 mM HEPES (pH 7.2–7.5), 10 mM MgCl_2_, 1 mM EGTA, 0.1 mM DTT and 0.01% (v/v) Brij^(R)^ 35. All assays were carried out at room temperature in triplicate or quadruplicate formats.

The percentage of inhibition by test compounds was calculated according to equation (1):

1 $$\mathrm{Percentage\; of\; inhibition}=\mathrm{100}\times (\frac{{\mu }_{{\rm{H}}}-T}{{\mu }_{{\rm{H}}}-{\mu }_{{\rm{L}}}})$$

where *T* is the value of the wells containing test compounds, and *μ*_H_ and *μ*_L_ are the mean values of the 0% and 100% inhibition control wells, respectively. The half maximal inhibitory concentration (IC_50_) of test compounds was calculated by fitting the data with the logistic equation using GraphPad Prism 5 (GraphPad Software, Inc., La Jolla, CA, USA) or XLfit (IDBS, Guildford, UK).

### Measurement of Inhibitory Activity of an ABL1 Inhibitor that Binds to the ATP Binding Site

Before addition to the assay plate, 3-fold concentrations of the His-ABL1 protein, Tb-SA and the biotinylated anti-His antibody were mixed in the assay buffer and incubated for over 1 h at room temperature. Several concentrations of test inhibitors dissolved in the assay buffer were dispensed in the assay plate. Subsequently, the ABL/antibody/Tb-SA premix was dispensed to each well and incubated for 120 min at room temperature. The reaction was initiated by addition of the assay buffer containing 13.5 nM BODIPY-dasatinib. The plate was incubated for 30 min at room temperature and the TR-FRET signal was measured using the EnVision Multilabel Plate Reader. The final concentrations of Tb-SA, biotinylated anti-His, ABL1 protein and BODIPY-dasatinib were 0.2, 0.4, 0.38 and 4.5 nM, respectively. The values of the 0% and 100% controls were the signals obtained in the absence and presence of 3 μM dasatinib, respectively.

### Measurement of Inhibitory Activity of IAP/Peptide Interaction

His-IAP proteins (XIAP, cIAP1 or cIAP2), FITC-Smac, Tb-SA and the biotinylated anti-His antibody (Life Technologies) were mixed in the assay buffer and incubated for over 1 h at room temperature before addition to the assay plate. Several concentrations of test inhibitors were dispensed in the assay plate and the protein-probe premix was dispensed to each well. All assays were carried out using 0.6 nM of IAP proteins. The concentrations of FITC-Smac were: 27 nM for XIAP, 12 nM for cIAP1 and 19 nM cIAP2. The final concentrations of Tb-SA and the biotinylated anti-His antibody were 0.2 and 0.4 nM, respectively. After 1 h incubation at room temperature, the TR-FRET signal was measured using the EnVision Multilabel Plate Reader. The values of the 0% and 100% controls were the signals obtained in the presence and absence of IAP proteins, respectively.

### Cell Viability Assay

Cell viability was determined using water-soluble tetrazolium WST-8 (4-[3-(2-methoxy-4-nitrophenyl)-2-(4-nitrophenyl)-2*H*-5-tetrazolio]-1,3-benzene disulfonate) for the spectrophotometric assay and conducted according to the manufacturer’s instructions (Dojindo, Tokyo, Japan). Cells were seeded at a concentration of 5 × 10^3^ cells per well in a 96-well culture plate, and treated with the indicated compounds for 48 h. The WST-8 reagent was added, and the cells were incubated for 0.5 h at 37 °C in a humidified atmosphere of 5% CO_2_. The absorbance at 450 nm of the medium was measured using the EnVision Multilabel Plate Reader.

### Washout experiment

Cells were seeded at a concentration of 2 × 10^6^ cells per 10-cm tissue culture dish and treated with the indicated compounds for the indicated times. Then, the cells were washed three times to remove the compounds, and incubated in drug-free medium for the indicated times.

### Statistical Analysis

Two-tailed Student’s *t*-test was used to determine the significance of differences among the experimental groups. Values of *P* < 0.05 were considered significant and the actual *P* values were presented.

## Electronic supplementary material

Supplementary information

## Data Availability

All data generated or analyzed during this study are included in this published article and its Supplementary Information files.

## References

[1] Rudkin CT, Hungerford DA, Nowell PC (1964). DNA Contents of Chromosome Ph1 and Chromosome 21 in Human Chronic Granulocytic Leukemia. Science.

[2] Rowley JD (1973). Letter: A new consistent chromosomal abnormality in chronic myelogenous leukaemia identified by quinacrine fluorescence and Giemsa staining. Nature.

[3] Konopka JB, Watanabe SM, Witte ON (1984). An alteration of the human c-abl protein in K562 leukemia cells unmasks associated tyrosine kinase activity. Cell.

[4] Shtivelman E, Lifshitz B, Gale RP, Canaani E (1985). Fused transcript of abl and bcr genes in chronic myelogenous leukaemia. Nature.

[5] Heisterkamp N, Stam K, Groffen J, de Klein A, Grosveld G (1985). Structural organization of the bcr gene and its role in the Ph’ translocation. Nature.

[6] Druker BJ (1996). Effects of a selective inhibitor of the Abl tyrosine kinase on the growth of Bcr-Abl positive cells. Nat. Med..

[7] Talpaz M (2006). Dasatinib in imatinib-resistant Philadelphia chromosome-positive leukemias. N. Engl. J. Med..

[8] Puttini M (2006). *In vitro* and *in vivo* activity of SKI-606, a novel Src-Abl inhibitor, against imatinib-resistant Bcr-Abl+ neoplastic cells. Cancer Res..

[9] Druker BJ (2008). Translation of the Philadelphia chromosome into therapy for CML. Blood.

[10] Demizu Y (2012). Design and synthesis of estrogen receptor degradation inducer based on a protein knockdown strategy. Bioorg. Med. Chem. Lett..

[11] Demizu Y (2016). Development of BCR-ABL degradation inducers via the conjugation of an imatinib derivative and a cIAP1 ligand. Bioorg. Med. Chem. Lett..

[12] Hattori T (2017). Simple and efficient knockdown of His-tagged proteins by ternary molecules consisting of a His-tag ligand, a ubiquitin ligase ligand, and a cell-penetrating peptide. Bioorg. Med. Chem. Lett..

[13] Itoh Y (2012). Double protein knockdown of cIAP1 and CRABP-II using a hybrid molecule consisting of ATRA and IAPs antagonist. Bioorg. Med. Chem. Lett..

[14] Itoh Y, Ishikawa M, Naito M, Hashimoto Y (2010). Protein knockdown using methyl bestatin-ligand hybrid molecules: design and synthesis of inducers of ubiquitination-mediated degradation of cellular retinoic acid-binding proteins. J. Am. Chem. Soc..

[15] Ohoka N (2014). Cancer cell death induced by novel small molecules degrading the TACC3 protein via the ubiquitin-proteasome pathway. Cell Death. Dis..

[16] Ohoka N (2017). SNIPER(TACC3) induces cytoplasmic vacuolization and sensitizes cancer cells to Bortezomib. Cancer Sci..

[17] Ohoka N (2017). *In Vivo* Knockdown of Pathogenic Proteins via Specific and Nongenetic Inhibitor of Apoptosis Protein (IAP)-dependent Protein Erasers (SNIPERs). J. Biol. Chem..

[18] Ohoka N, Shibata N, Hattori T, Naito M (2016). Protein Knockdown Technology: Application of Ubiquitin Ligase to Cancer Therapy. Curr. Cancer Drug Targets.

[19] Okuhira K (2013). Development of hybrid small molecules that induce degradation of estrogen receptor-alpha and necrotic cell death in breast cancer cells. Cancer Sci..

[20] Okuhira K (2011). Specific degradation of CRABP-II via cIAP1-mediated ubiquitylation induced by hybrid molecules that crosslink cIAP1 and the target protein. FEBS Lett..

[21] Okuhira K (2017). Targeted Degradation of Proteins Localized in Subcellular Compartments by Hybrid Small Molecules. Mol. Pharmacol..

[22] Shibata N (2017). Development of protein degradation inducers of oncogenic BCR-ABL protein by conjugation of ABL kinase inhibitors and IAP ligands. Cancer Sci..

[23] Shibata N (2018). Development of Protein Degradation Inducers of Androgen Receptor by Conjugation of Androgen Receptor Ligands and Inhibitor of Apoptosis Protein Ligands. J. Med. Chem..

[24] Shimokawa K (2017). Targeting the Allosteric Site of Oncoprotein BCR-ABL as an Alternative Strategy for Effective Target Protein Degradation. ACS Med. Chem. Lett..

[25] Tomoshige S, Naito M, Hashimoto Y, Ishikawa M (2015). Degradation of HaloTag-fused nuclear proteins using bestatin-HaloTag ligand hybrid molecules. Org. Biomol. Chem..

[26] Bondeson DP (2015). Catalytic *in vivo* protein knockdown by small-molecule PROTACs. Nat. Chem. Biol..

[27] Buckley DL (2015). HaloPROTACS: Use of Small Molecule PROTACs to Induce Degradation of HaloTag Fusion Proteins. ACS Chem. Biol..

[28] Chan KH, Zengerle M, Testa A, Ciulli A (2018). Impact of Target Warhead and Linkage Vector on Inducing Protein Degradation: Comparison of Bromodomain and Extra-Terminal (BET) Degraders Derived from Triazolodiazepine (JQ1) and Tetrahydroquinoline (I-BET726) BET Inhibitor Scaffolds. J. Med. Chem..

[29] Gadd MS (2017). Structural basis of PROTAC cooperative recognition for selective protein degradation. Nat. Chem. Biol..

[30] Lai AC (2016). Modular PROTAC Design for the Degradation of Oncogenic BCR-ABL. Angew. Chem. Int. Ed. Engl..

[31] Lu J (2015). Hijacking the E3 Ubiquitin Ligase Cereblon to Efficiently Target BRD4. Chem. Biol..

[32] Puppala D, Lee H, Kim KB, Swanson HI (2008). Development of an aryl hydrocarbon receptor antagonist using the proteolysis-targeting chimeric molecules approach: a potential tool for chemoprevention. Mol. Pharmacol..

[33] Raina K (2016). PROTAC-induced BET protein degradation as a therapy for castration-resistant prostate cancer. Proc. Natl. Acad. Sci. USA.

[34] Rodriguez-Gonzalez A (2008). Targeting steroid hormone receptors for ubiquitination and degradation in breast and prostate cancer. Oncogene.

[35] Sakamoto KM (2003). Development of Protacs to target cancer-promoting proteins for ubiquitination and degradation. Mol. Cell. Proteomics.

[36] Schneekloth AR, Pucheault M, Tae HS, Crews CM (2008). Targeted intracellular protein degradation induced by a small molecule: En route to chemical proteomics. Bioorg. Med. Chem. Lett..

[37] Schneekloth JS (2004). Chemical genetic control of protein levels: selective *in vivo* targeted degradation. J. Am. Chem. Soc..

[38] Toure M, Crews CM (2016). Small-Molecule PROTACS: New Approaches to Protein Degradation. Angew. Chem. Int. Ed. Engl..

[39] Winter GE (2015). DRUG DEVELOPMENT. Phthalimide conjugation as a strategy for *in vivo* target protein degradation. Science.

[40] Zengerle M, Chan KH, Ciulli A (2015). Selective Small Molecule Induced Degradation of the BET Bromodomain Protein BRD4. ACS Chem. Biol..

[41] Zhou B (2018). Discovery of a Small-Molecule Degrader of Bromodomain and Extra-Terminal (BET) Proteins with Picomolar Cellular Potencies and Capable of Achieving Tumor Regression. J. Med. Chem..

[42] Winter, G. E. *et al*. BET Bromodomain Proteins Function as Master Transcription Elongation Factors Independent of CDK9 Recruitment. *Mol. Cell* **67** 5-18 e19 (2017). 10.1016/j.molcel.2017.06.004 PMC5663500 28673542

[43] Burslem, G. M. *et al*. The Advantages of Targeted Protein Degradation Over Inhibition: An RTK Case Study. *Cell Chem. Biol*. (2017). 10.1016/j.chembiol.2017.09.009 PMC5831399 29129716

[44] Shah NP (2004). Overriding imatinib resistance with a novel ABL kinase inhibitor. Science.

[45] Weisberg E (2010). Discovery of a small-molecule type II inhibitor of wild-type and gatekeeper mutants of BCR-ABL, PDGFRalpha, Kit, and Src kinases: novel type II inhibitor of gatekeeper mutants. Blood.

[46] Weisberg E (2010). Smac mimetics: implications for enhancement of targeted therapies in leukemia. Leukemia.

[47] Galdeano C (2014). Structure-guided design and optimization of small molecules targeting the protein-protein interaction between the von Hippel-Lindau (VHL) E3 ubiquitin ligase and the hypoxia inducible factor (HIF) alpha subunit with *in vitro* nanomolar affinities. J. Med. Chem..

[48] Fischer ES (2014). Structure of the DDB1-CRBN E3 ubiquitin ligase in complex with thalidomide. Nature.

[49] Brave M (2008). Sprycel for chronic myeloid leukemia and Philadelphia chromosome-positive acute lymphoblastic leukemia resistant to or intolerant of imatinib mesylate. Clin. Cancer Res..

[50] Cromm PM, Crews CM (2017). Targeted Protein Degradation: from Chemical Biology to Drug Discovery. Cell Chem. Biol..

[51] Salami J, Crews CM (2017). Waste disposal-An attractive strategy for cancer therapy. Science.

[52] Mahon FX (2010). Discontinuation of imatinib in patients with chronic myeloid leukaemia who have maintained complete molecular remission for at least 2 years: the prospective, multicentre Stop Imatinib (STIM) trial. Lancet Oncol..

[53] Etienne G (2017). Long-Term Follow-Up of the French Stop Imatinib (STIM1) Study in Patients With Chronic Myeloid Leukemia. J. Clin. Oncol..

[54] Takahashi, N. *et al*. Deeper molecular response is a predictive factor for treatment-free remission after imatinib discontinuation in patients with chronic phase chronic myeloid leukemia: the JALSG-STIM213 study. *Int. J. Hematol*. 10.1007/s12185-017-2334-x (2017). 10.1007/s12185-017-2334-x 28929332

[55] Baccarani M (2013). European LeukemiaNet recommendations for the management of chronic myeloid leukemia: 2013. Blood.

[56] Nikolovska-Coleska Z (2004). Development and optimization of a binding assay for the XIAP BIR3 domain using fluorescence polarization. Anal. Biochem..

[57] Vetter ML (2014). Fluorescent visualization of Src by using dasatinib-BODIPY. Chembiochem.

[58] Kishi K (1985). A new leukemia cell line with Philadelphia chromosome characterized as basophil precursors. Leuk. Res..

