# Supplementary material for: Pharmacological difference between degrader and inhibitor against oncogenic BCR-ABL kinase
Source: Sci Rep. 2018 Sep 10;8:13549. doi: 10.1038/s41598-018-31913-5 (PMC6131351; doi:10.1038/s41598-018-31913-5)

Supplementary Information

Pharmacological difference between degrader and inhibitor against oncogenic BCR-ABL kinase

Norihito Shibata[1], Kenichiro Shimokawa[2], Katsunori Nagai[2, 3], Nobumichi Ohoka[1], Takayuki Hattori[1], Naoki Miyamoto[2], Osamu Ujikawa[2, 3], Tomoya Sameshima[2], Hiroshi Nara[2,4], Nobuo Cho[2,5], and Mikihiko Naito[1, *]

[1]Divisions of Molecular Target and Gene Therapy Products, National Institute of Health Sciences, 3-25-26 Tonomachi, Kawasaki-ku, Kawasaki-shi, Kanagawa 210-9501, Japan.

[2]Pharmaceutical Research Division, Takeda Pharmaceutical Co. Ltd., Kanagawa, Japan.
[3]Present address: Axcelead Drug Discovery Partners, Inc., Kanagawa, Japan.
[4]Present address: The Pharmaceutical Society of Japan, Tokyo, Japan. [5]Present address: Drug Discovery Chemistry Platform Unit (Wako branch), RIKEN Center for Life Science Technologies, Saitama, Japan.
[*]Correspondence and requests for materials should be addressed to Dr. Mikihiko Naito, Divisions of Molecular Target and Gene Therapy Products, National Institute of Health Sciences, 3-25-26 Tonomachi, Kawasaki-ku, Kawasaki-shi, Kanagawa 210-9501, Japan. Tel: +81-44-270-6533. Fax: +81-44-270-6534. E-mail: miki-naito@nihs.go.jp

**Doc. S1**	The chemical synthesis and physicochemical data
**Scheme S1**	Synthesis of DAS-VHL and HG-VHL
**Scheme S2**	Synthesis of DAS-CRBN and HG-CRBN
**Scheme S3**	Synthesis of DAS-meIAP
**Figure S1**	Growth inhibitory effect of DAS-IAP, DAS-meIAP, and dasatinib in BCR-ABL negative cells
**Figure S2**	Original images of western blot analysis

Doc. S1. The chemical synthesis and physicochemical data.

General remarks

The proton nuclear magnetic resonance (^{1}H NMR) spectra were determined on a Bruker AVANCE II (300 MHz or 400 MHz) spectrometer. Chemical shifts were reported in parts per million (ppm) downfield from tetramethylsilane (δ) as the internal standard in deuterated solvent and coupling constants (*J*) are in Hertz (Hz). The following abbreviations are used for spin multiplicity: s = singlet, d = doublet, t = triplet, q = quartet, quin = quintet, dd = doublet of doublet, dt = doublet of triplet, qd = quartet of doublet, dquin = doublet of quintet, m = multiplet, and br s = broad singlet. Reaction progress was determined by thin layer chromatography (TLC) analysis on silica gel 60 F_{254} plates (Merck) or NH TLC plate (Fuji Silysia Chemical Ltd., Aichi, Japan). Column chromatography was performed with a silica gel column [(Merck Kieselgel 60, 70–230 mesh, Merck) or (Chromatorex® NH-DM1020, 100–200 mesh, Fuji Silysia Chemical Ltd.)], or with prepacked Purif-Pack columns [silica gel or NH (3-aminopropyl-functionalized) silica gel, particle size: 60 µm, Fuji Silysia Chemical Ltd.]. Low-resolution mass spectra (MS) were acquired using an Agilent LC/MS system (Agilent1200SL/Agilent6130MS, Agilent1200SL/Agilent1956MS or Agilent1200SL/Agilent6110MS), Shimadzu UFLC/MS (Shimazu LC-20AD/LCMS-2020) operating in electron spray ionization mode (ESI+). The column used was an L-column 2 ODS (3.0 × 50 mm I.D., 3 µm, CERI, Japan) with a temperature of 40 °C and a flow rate of 1.2 or 1.5 mL/min or an Waters X-Bridge C18 (4.6 × 50 mm I.D., 3.5 µm) with a temperature of 40 °C and a flow rate of 2.0 mL/min. Mobile Phase: Condition 1: Mobile phases A and B under an acidic condition were 0.05% TFA in water and 0.05% TFA in MeCN, respectively. The ratio of mobile phase B was increased linearly from 5% to 90% over 0.9 min, 90% over the next 1.1 min, or the ratio of mobile phase B was increased linearly from 5% to 100% over 1.6 min, 100% over the next 1.4 min, or the ratio of mobile phase B was increased linearly from 5% to 100% over 3.0 min, 100% over the next 1.0 min. Condition 2: Mobile phases A and B under a neutral condition were a mixture of 5 mmol/L $AcONH_4$ and MeCN (9:1, v/v) and a mixture of 5 mmol/L $AcONH_4$ and MeCN (1:9, v/v), respectively. The ratio of mobile phase B was increased linearly from 5% to 90% over 0.9 min, 90% over the next 1.1 min. The purities of all compounds tested in biological systems were assessed as being > 95% using elemental analysis or analytical HPLC. Purity data were collected by HPLC with NQAD (Nano Quality Analyte Detector) or Corona CAD (Charged Aerosol Detector). The column was an L-column 2 ODS (30 × 2.1 mm I.D., CERI, Japan) or a Capcell Pak C18AQ (50 mm × 3.0 mm I.D., Shiseido,

Japan) with a temperature of 50 °C and a flow rate of 0.5 mL/min. Mobile phases A and B under a neutral condition were a mixture of 50 mmol/L ammonium acetate, water and acetonitrile (1:8:1, v/v/v) and a mixture of 50 mmol/L ammonium acetate and MeCN (1:9, v/v), respectively. The ratio of mobile phase B was increased linearly from 5% to 95% over 3 min, 95% over the next 1 min. All commercially available solvents and reagents were used without further purification. Yields were not optimized.

Abbreviations are used as follows: CD_3OD, deuterated methanol; DIPEA, *N,N'*-diisopropylethylamine; DMAP, 4-dimethylaminopyridine; DMF, *N,N*-dimethylformamide; DMSO-d_6, dimethyl sulfoxide-d_6; EDC, 1-(3-(dimethylamino)propyl)-3-ethylcarbodiimide; EtOAc, ethyl acetate; Et_3N, triethylamine; HATU, 1-[bis(dimethylamino)methylene]-1*H*-1,2,3-triazolo[4,5-*b*]pyridinium 3-oxid hexafluorophosphate; HOBt, 1-hydroxybenzotriazole; IPE, diisopropyl ether; MeCN, acetonitrile; MeOH, methanol; MNBA, 2-methyl-6-nitrobenzoic anhydride; NMP, *N*-methylpyrrolidone; TBDMSCl, *tert*-butyldimethylchlorosilane; THF, tetrahydrofuran.

Experimental procedures

***N*-((2-(2-(Carboxymethoxy)ethoxy)ethoxy)acetyl)-3-methyl-L-valyl-(4*R*)-4-hydroxy-*N*-(4-(4-methyl-1,3-thiazol-5-yl)benzyl)-L-prolinamide (3).**

A mixture of 2,2'-((oxybis(ethane-2,1-diyl))bis(oxy))diacetic acid (**1**) (0.39 g, 1.8 mmol), (2*S*,4*R*)-1-((*S*)-2-amino-3,3-dimethylbutanoyl)-4-hydroxy-*N*-(4-(4-methylthiazol-5-yl)benzyl)pyrrolidine-2-carboxamide (**2**)[1] (0.25 g, 0.58 mmol), HOBt (0.11 g, 0.81 mmol), and EDC (0.15 mL, 0.86 mmol) in THF (5 mL) and DMF (0.5 mL) was stirred at room temperature for 1 h. To the mixture was added 1 N NaOH aq. (4 mL), and the mixture was stirred at room temperature for 20 min. After the mixture was acidified with 1 N HCl aq. (5 mL), the mixture was diluted with EtOAc, washed with brine, dried over Na_2SO_4, and concentrated in vacuo. The residue was purified by column chromatography (silica gel, eluted with 0–100% MeOH in EtOAc) to give the title compound (316 mg, 86%) as a colorless solid. ^{1}H NMR (300 MHz, CD_3OD) δ 0.98–1.08 (9H, m), 2.02–2.33 (2H, m), 2.47 (3H, s), 3.59–4.18 (14H, m), 4.30–4.75 (5H, m), 7.37–7.52 (4H, m), 8.87 (1H, s); MS m/z 635.3 $[M+H]^+$.

***N*-((2-(2-(Carboxymethoxy)ethoxy)ethoxy)acetyl)-3-methyl-L-valyl-(4*R*)-4-((*tert*-butyl(dimethyl)silyl)oxy)-*N*-(4-(4-methyl-1,3-thiazol-5-yl)benzyl)-L-prolinamide (4).**

A mixture of **3** (0.10 g, 0.16 mmol), imidazole (54 mg, 0.79 mmol) and TBDMSCl (71 mg,

0.47 mmol) in THF (1 mL) was stirred at room temperature overnight. To the mixture was added 1 N NaOH aq. (0.5 mL), and the mixture was stirred at room temperature for 1 h. After the mixture was acidified with 1 N HCl aq. (1 mL), the mixture was diluted with EtOAc, washed with brine, dried over Na_2SO_4, and concentrated in vacuo. The residue was purified by column chromatography (silica gel, eluted with 0–100% MeOH in EtOAc) to give the title compound (86 mg, 73%) as a colorless solid. ^{1}H NMR (300 MHz, CD_3OD) δ 0.11 (6H, s), 0.87 (9H, s), 1.03 (9H, s), 2.02–2.39 (2H, m), 2.47 (3H, s), 3.60–4.16 (14H, m), 4.33–4.75 (5H, m), 7.37–7.50 (4H, m), 8.87 (1H, s); MS *m/z* 749.4 $[M+H]^+$.

***N*-((2-(2-(2-(4-(6-((5-((2-Chloro-6-methylphenyl)carbamoyl)-1,3-thiazol-2-yl)amino)-2-methylpyrimidin-4-yl)piperazin-1-yl)-2-oxoethoxy)ethoxy)ethoxy)acetyl)-3-methyl-L-valyl-(4*R*)-4-hydroxy-*N*-(4-(4-methyl-1,3-thiazol-5-yl)benzyl)-L-prolinamide (6, DAS-VHL).**

A mixture of **3** (57.2 mg, 0.09 mmol), *N*-(2-chloro-6-methylphenyl)-2-((2-methyl-6-(piperazin-1-yl)pyrimidin-4-yl)amino)thiazole-5-carboxamide (**5**)[2] (40 mg, 0.09 mmol), HATU (44.5 mg, 0.12 mmol), and DIPEA (83 μL, 0.48 mmol) in MeCN (1.5 mL) was stirred at room temperature overnight. The mixture was diluted with EtOAc, washed with sat. $NaHCO_3$ aq. and brine, dried over Na_2SO_4, and concentrated in vacuo. The residue was purified by column chromatography (silica gel, eluted with 60–100% EtOAc in hexane) to give the title compound (33.0 mg, 35%) as a colorless gum. ^{1}H NMR (300 MHz, CD_3OD) δ 0.88–1.01 (10H, m), 1.93–2.05 (1H, m), 2.06–2.18 (1H, m), 2.22 (3H, s), 2.31–2.43 (6H, m), 3.39–4.07 (19H, m), 4.12–4.62 (7H, m), 5.91 (1H, s), 7.07–7.41 (7H, m), 8.05 (1H, s), 8.69–8.80 (1H, m); MS *m/z* 1060.4 $[M+Na]^+$; Purity 100% (HPLC).

***N*-((2-(2-(2-((5-(3-((4-((4-Ethylpiperazin-1-yl)methyl)-3-(trifluoromethyl)phenyl)carbamoyl)phenyl)[1,3]thiazolo[5,4-*b*]pyridin-2-yl)amino)-2-oxoethoxy)ethoxy)ethoxy)acetyl)-3-methyl-L-valyl-(4*R*)-4-((*tert*-butyl(dimethyl)silyl)oxy)-*N*-(4-(4-methyl-1,3-thiazol-5-yl)benzyl)-L-prolinamide (8).**

To a mixture of **4** (86 mg, 0.11 mmol), 3-(2-aminothiazolo[5,4-*b*]pyridin-5-yl)-*N*-(4-((4-ethylpiperazin-1-yl)methyl)-3-(trifluoromethyl)phenyl)benzamide (**7**)[2] (68 mg, 0.13 mmol), Et_3N (64 μL, 0.46 mmol), and DMAP (15 mg, 0.12 mmol) in DMF (1 mL) was added MNBA (79 mg, 0.23 mmol) at 0 °C. The mixture was stirred at 0 °C for 2 h. The mixture was diluted with EtOAc, washed with 5% Na_2CO_3 aq. and brine, dried over Na_2SO_4, and concentrated in vacuo. The residue was purified by column chromatography (NH silica gel, eluted with 0–20%

MeOH in EtOAc) to give the title compound (86 mg, 59%) as a colorless solid. ^{1}H NMR (300 MHz, CD_3OD) δ 0.02–0.11 (6H, m), 0.75–0.86 (9H, m), 0.96–1.05 (9H, m), 1.12 (3H, t, J = 7.2 Hz), 2.02–2.23 (2H, m), 2.37–2.72 (13H, m), 3.52–4.78 (21H, m), 7.25–7.44 (4H, m), 7.64 (1H, t, J = 7.7 Hz), 7.77 (1H, d, J = 8.4 Hz), 7.94–8.06 (3H, m), 8.09–8.14 (1H, m), 8.16 (1H, s), 8.23–8.35 (1H, m), 8.64 (1H, s), 8.73–8.89 (1H, m); MS *m/z* 1271.6 $[M+H]^+$.

***N*-((2-(2-(2-((5-(3-((4-((4-Ethylpiperazin-1-yl)methyl)-3-(trifluoromethyl)phenyl)carbamoyl)phenyl)[1,3]thiazolo[5,4-*b*]pyridin-2-yl)amino)-2-oxoethoxy)ethoxy)ethoxy)acetyl)-3-methyl-L-valyl-(4*R*)-4-hydroxy-*N*-(4-(4-methyl-1,3-thiazol-5-yl)benzyl)-L-prolinamide (9, HG-VHL).**

A mixture of **8** (85 mg, 0.067 mmol) and 0.1 M TBAF in THF (2.0 mL, 0.20 mmol) was stirred at room temperature for 1 h. The mixture was diluted with EtOAc, washed with 5% Na_2CO_3 aq. and brine, dried over Na_2SO_4, and concentrated in vacuo. The residue was purified by column chromatography (silica gel, eluted with 0–100% MeOH in EtOAc), and then (NH silica gel, eluted with 0–40% EtOAc in hexane) to give the title compound (45.0 mg, 58%) as a colorless solid. ^{1}H NMR (300 MHz, CD_3OD) δ 0.95–1.05 (9H, m), 1.11 (3H, t, J = 7.2 Hz), 2.03–2.28 (2H, m), 2.37–2.79 (13H, m), 3.63–3.92 (12H, m), 3.98–4.78 (9H, m), 7.26–7.44 (4H, m), 7.64 (1H, t, J = 7.7 Hz), 7.77 (1H, d, J = 8.5 Hz), 7.92–8.14 (4H, m), 8.16 (1H, s), 8.27 (1H, d, J = 7.5 Hz), 8.63 (1H, s), 8.75–8.86 (1H, m); MS *m/z* 1157.4 $[M+H]^+$; Purity 100% (HPLC).

(2-(2-(2-((2-(2,6-Dioxopiperidin-3-yl)-1,3-dioxo-2,3-dihydro-1*H*-isoindol-4-yl)amino)-2-oxoethoxy)ethoxy)ethoxy)acetic acid (11).

To a solution of **1** (465 mg, 1.46 mmol) and oxalyl chloride (0.511 mL, 5.86 mmol) in THF (1 mL) was added DMF (1 drop) at room temperature. The mixture was stirred at room temperature for 1 h and the mixture was concentrated in vacuo. The residue in THF (1 mL) was added to a solution of 4-amino-2-(2,6-dioxopiperidin-3-yl)isoindoline-1,3-dione (**10**) (200 mg, 0.73 mmol) in THF (1 mL). The mixture was stirred at 60 °C for 3 h. The mixture was quenched with 1N HCl aq. at room temperature and extracted with EtOAc. The organic layer was separated, washed with water and brine, dried over $MgSO_4$, and concentrated in vacuo. The residue was purified by column chromatography (silica gel, eluted with 0–30% MeOH in EtOAc). The desired fractions were concentrated in vacuo and the resulting solid was washed with IPE–EtOAc and dried in vacuo to give the title compound (317 mg, 91%) as a pale yellow solid. ^{1}H NMR (300 MHz, DMSO-d_6) δ 2.00–2.16 (1H, m), 2.57–2.65 (1H, m), 2.80–3.01 (1H,

m), 3.54–3.60 (4H, m), 3.62–3.82 (5H, m), 3.98 (2H, s), 4.21 (2H, s), 5.16 (1H, dd, *J* = 13.0, 5.0 Hz), 7.63 (1H, d, *J* = 7.4 Hz), 7.87 (1H, t, *J* = 7.8 Hz), 8.73 (1H, d, *J* = 8.3 Hz), 10.34–10.40 (1H, m), 11.08–11.22 (1H, m), 12.56 (1H, br s); MS *m/z* 478.0 $[M+H]^+$.

***N*-(2-Chloro-6-methylphenyl)-2-((6-(4-((2-(2-(2-((2-(2,6-dioxopiperidin-3-yl)-1,3-dioxo-2,3-dihydro-1*H*-isoindol-4-yl)amino)-2-oxoethoxy)ethoxy)ethoxy)acetyl)piperazin-1-yl)-2-methylpyrimidin-4-yl)amino)-1,3-thiazole-5-carboxamide (12, DAS-CRBN).**

A mixture of **11** (55.8 mg, 0.13 mmol), **5** (55.8 mg, 0.13 mmol), HATU (62.1 mg, 0.16 mmol), and DIPEA (0.044 mL, 0.25 mmol) in DMF (0.5 mL) was stirred at room temperature for 3 h. The mixture was quenched with water at room temperature and extracted with EtOAc. The organic layer was separated, washed with water and brine, dried over $MgSO_4$, and concentrated in vacuo. The residue was purified by column chromatography (silica gel, eluted with 0–30% MeOH in EtOAc) to give the title compound (35.0 mg, 31%) as a colorless solid. ^1H NMR (300 MHz, CD_3OD) δ 2.12–2.26 (1H, m), 2.36 (3H, s), 2.70–3.03 (4H, m), 3.62–3.95 (18H, m), 4.19 (2H, s), 4.28 (2H, s), 5.18 (1H, dd, *J* = 12.3, 5.6 Hz), 6.08–6.25 (1H, m), 7.24–7.44 (3H, m), 7.48–7.55 (1H, m), 7.71 (1H, t, *J* = 7.4 Hz), 8.19 (1H, br s), 8.70 (1H, d, *J* = 8.3 Hz); MS *m/z* 903.2 $[M+H]^+$; Purity 100% (HPLC).

3-(2-(((2-(2-(2-((2-(2,6-Dioxopiperidin-3-yl)-1,3-dioxo-2,3-dihydro-1*H*-isoindol-4-yl)amino)-2-oxoethoxy)ethoxy)ethoxy)acetyl)amino)[1,3]thiazolo[5,4-*b*]pyridin-5-yl)-*N*-(4-((4-ethylpiperazin-1-yl)methyl)-3-(trifluoromethyl)phenyl)benzamide (13, HG-CRBN).

A mixture of **11** (60 mg, 0.13 mmol), **7** (67.9 mg, 0.13 mmol), HATU (62.1 mg, 0.16 mmol), and DIPEA (0.044 mL, 0.25 mmol) in DMF (0.5 mL) was stirred at room temperature overnight. The mixture was poured into water at room temperature and extracted with EtOAc. The organic layer was separated, washed with water and brine, dried over $MgSO_4$, and concentrated in vacuo. The residue was purified by column chromatography (silica gel, eluted with 0–30% MeOH in EtOAc and then NH silica gel, eluted with 0–20% MeOH in EtOAc) to give the title compound (18 mg, 14%) as a colorless solid. ^1H NMR (300 MHz, CD_3OD) δ 1.02 (3H, t, *J* = 7.2 Hz), 2.05–2.22 (1H, m), 2.32–2.56 (9H, m), 2.59–2.83 (3H, m), 3.58 (2H, s), 3.63–3.93 (8H, m), 4.07–4.13 (2H, m), 4.18–4.25 (2H, m), 4.42–4.57 (1H, m), 5.00 (1H, dd, *J* = 12.2, 5.2 Hz), 7.20 (1H, d, *J* = 7.2 Hz), 7.38 (1H, t, *J* = 7.9 Hz), 7.49–7.59 (1H, m), 7.69 (1H, d, *J* = 8.7 Hz), 7.81–7.95 (4H, m), 8.05–8.16 (2H, m), 8.37 (1H, d, *J* = 8.6 Hz), 8.48 (1H, s); MS *m/z* 1000.3 $[M+H]^+$; Purity 100% (HPLC).

Methyl (2-(2-(2-(3-((2-((2*S*)-1-((2*S*)-2-((*N*-(*tert*-butoxycarbonyl)-*N*-methyl-L-alanyl)(methyl)amino)-2-cyclohexylacetyl)pyrrolidin-2-yl)-1,3-thiazol-4-yl)carbonyl)phenoxy)ethoxy)ethoxy)ethoxy)acetate (16)

A mixture of methyl 2-(2-(2-(2-(tosyloxy)ethoxy)ethoxy)ethoxy)acetate (**15**)[2] (61.4 mg, 0.16 mmol), tert-butyl ((S)-1-(((S)-1-cyclohexyl-2-((S)-2-(4-(3-hydroxybenzoyl)thiazol-2-yl)pyrrolidin-1-yl)-2-oxoethyl)(methyl)amino)-1-oxopropan-2-yl)(methyl)carbamate (**14**)[3] (100 mg, 0.16 mmol), and K_2CO_3 (33.8 mg, 0.24 mmol) in DMF (3 mL) was stirred at 50 °C overnight. After cooling, the mixture was diluted with EtOAc, washed with water and brine, dried over Na_2SO_4, and concentrated in vacuo. The residue was purified by column chromatography (NH silica gel, eluted with 10–30% EtOAc in hexane) to give the title compound **16** (36.0 mg, 0.044 mmol, 27.0%) as a colorless oil. ^{1}H NMR (300 MHz, $CDCl_3$) δ 0.83–1.19 (5H, m), 1.29 (3H, d, *J* = 5.1 Hz), 1.47 (9H, s), 1.57–1.78 (5H, m), 2.06–2.33 (3H, m), 2.38-2.52 (1H, m), 2.72–2.79 (3H, m), 3.10 (3H, s), 3.68–3.75 (12H, m), 3.79–3.94 (4H, m), 4.16–4.22 (4H, m), 5.13 (2H, d, *J* = 9.8 Hz), 5.41–5.57 (1H, m), 7.16 (1H, dd, *J* = 8.9, 2.7 Hz), 7.37 (1H, t, *J* = 8.0 Hz), 7.67–7.72 (1H, m), 7.76–7.87 (1H, m), 8.11 (1H, s). MS *m*/*z* 817.4 $(M+H)^+$.

(2-(2-(2-(3-((2-((2*S*)-1-((2*S*)-2-((*N*-(*tert*-Butoxycarbonyl)-*N*-methyl-LL-alanyl)(methyl)amino)-2-cyclohexylacetyl)pyrrolidin-2-yl)-1,3-thiazol-4-yl)carbonyl)phenoxy)ethoxy)ethoxy)ethoxy)acetic acid (17)

To a solution of **16** (36 mg, 0.04 mmol) in THF (0.5 mL)–MeOH (0.5 mL)–water (0.5 mL) was added 4M LiOH aq. (0.033 mL, 0.13 mmol) at room temperature. The mixture was stirred at room temperature for 30 min. The mixture was acidified with 1M HCl aq. (1.7 mL), diluted with EtOAc, washed with brine, dried over Na_2SO_4, and concentrated in vacuo to give the title compound **17** (35.3 mg, 0.044 mmol, 100% quant.) as a colorless gum. ^{1}H NMR (300 MHz, $CDCl_3$) δ 0.90–1.21 (5H, m), 1.29 (3H, d, *J* = 6.8 Hz), 1.47 (9H, s), 1.55–1.75 (5H, m), 2.15–2.33 (3H, m), 2.36–2.52 (1H, m), 2.76 (3H, s), 3.10 (3H, s), 3.68–3.77 (10H, m), 3.85–3.92 (3H, m), 4.14 (2H, s), 4.18–4.24 (2H, m, *J* = 4.5 Hz), 5.13 (2H, d, *J* = 10.3 Hz), 5.42–5.62 (1H, m), 7.16 (1H, dd, *J* = 7.8, 2.2 Hz), 7.38 (1H, t, *J* = 7.9 Hz), 7.70–7.84 (2H, m), 8.14 (1H, s). MS *m*/*z* 803.4 $(M+H)^+$.

tert-Butyl ((2*S*)-1-(((1*S*)-2-((2*S*)-2-(4-(3-(2-(2-(2-(2-(4-(6-((5-((2-chloro-6-methylphenyl)carbamoyl)-1,3-thiazol-2-yl)amino)-2-methylpyrimidin-4-yl)piperazin-1-

yl)-2-oxoethoxy)ethoxy)ethoxy)ethoxy)benzoyl)-1,3-thiazol-2-yl)pyrrolidin-1-yl)-1-cyclohexyl-2-oxoethyl)(methyl)amino)-1-oxopropan-2-yl)methylcarbamate (19)

A mixture of *N*-(2-Chloro-6-methylphenyl)-2-((2-methyl-6-(piperazin-1-yl)pyrimidin-4-yl)amino)thiazole-5-carboxamide **(18)** (19.4 mg, 0.04 mmol), **17** (35 mg, 0.04 mmol), HOBt (7.1 mg, 0.05 mmol), and EDC (9.18 μL, 0.05 mmol) in DMF (1.5 mL) was stirred at room temperature overnight. The mixture was diluted with EtOAc, washed with 5% $NaHCO_3$ aq. and brine, dried over Na_2SO_4, and concentrated in vacuo. The residue was purified by column chromatography (Silica gel, eluted with 0–20% MeOH in EtOAc) to give the title compound **19** (50.0 mg, 0.041 mmol, 93%) as a pale yellow amorphous solid. This was used for the next reaction without further purification. MS *m*/*z* 1228.4 $(M+H)^+$.

***N*-(2-Chloro-6-methylphenyl)-2-((6-(4-((2-(2-(2-(3-((2-((2*S*)-1-((2*S*)-2-cyclohexyl-2-(methyl(*N*-methyl-L-alanyl)amino)acetyl)pyrrolidin-2-yl)-1,3-thiazol-4-yl)carbonyl)phenoxy)ethoxy)ethoxy)ethoxy)acetyl)piperazin-1-yl)-2-methylpyrimidin-4-yl)amino)-1,3-thiazole-5-carboxamide (20, DAS-meIAP)**

A mixture of **19** (50 mg, 0.04 mmol) and TFA (2 mL) was stirred at room temperature for 3 h. After the mixture was concentrated in vacuo, the residue was dissolved in EtOAc–-IPA (4:1), washed with sat. $NaHCO_3$ aq. and brine, dried over Na_2SO_4, and concentrated in vacuo. The residue was purified by column chromatography (NH Silica gel, eluted with 0–10% MeOH in EtOAc) to give the title compound **20** (27.0 mg, 0.024 mmol, 98.8 %) as a colorless amorphous solid. ^{1}H NMR (300 MHz, CD_3OD) δ 0.87–1.05 (2H, m), 1.11–1.24 (6H, m), 1.47–1.81 (6H, m), 2.22–2.35 (9H, m), 2.43 (3H, s), 3.00 (3H, s), 3.60–3.71 (19H, m), 3.83–3.90 (3H, m), 4.13–4.21 (2H, m), 4.27 (2H, s), 5.21 (1H, d, *J* = 11.0 Hz), 5.38–5.47 (1H, m, *J* = 3.2 Hz), 5.94 (1H, s), 7.13–7.20 (1H, m), 7.25 (2H, s), 7.36 (2H, d, *J* = 7.9 Hz), 7.62–7.73 (2H, m), 8.10–8.17 (1H, m), 8.26 (1H, s). MS *m*/*z* 1128.6 $(M+H)^+$. Purity 100% (HPLC).

References

1 Buckley, D. L. *et al*. HaloPROTACS: Use of Small Molecule PROTACs to Induce Degradation of HaloTag Fusion Proteins. *ACS Chem. Biol.* **10**, 1831-1837 (2015).

2 Shibata, N. *et al*. Development of protein degradation inducers of oncogenic BCR-ABL protein by conjugation of ABL kinase inhibitors and IAP ligands. *Cancer Sci.* **108**, 1657-1666 (2017).

3 Ohoka, N. *et al*. In Vivo Knockdown of Pathogenic Proteins via Specific and Nongenetic Inhibitor of Apoptosis Protein (IAP)-dependent Protein Erasers (SNIPERs). *J. Biol. Chem.* **292**, 4556-4570 (2017).

Scheme S1. Synthesis of DAS-VHL and HG-VHL[a]

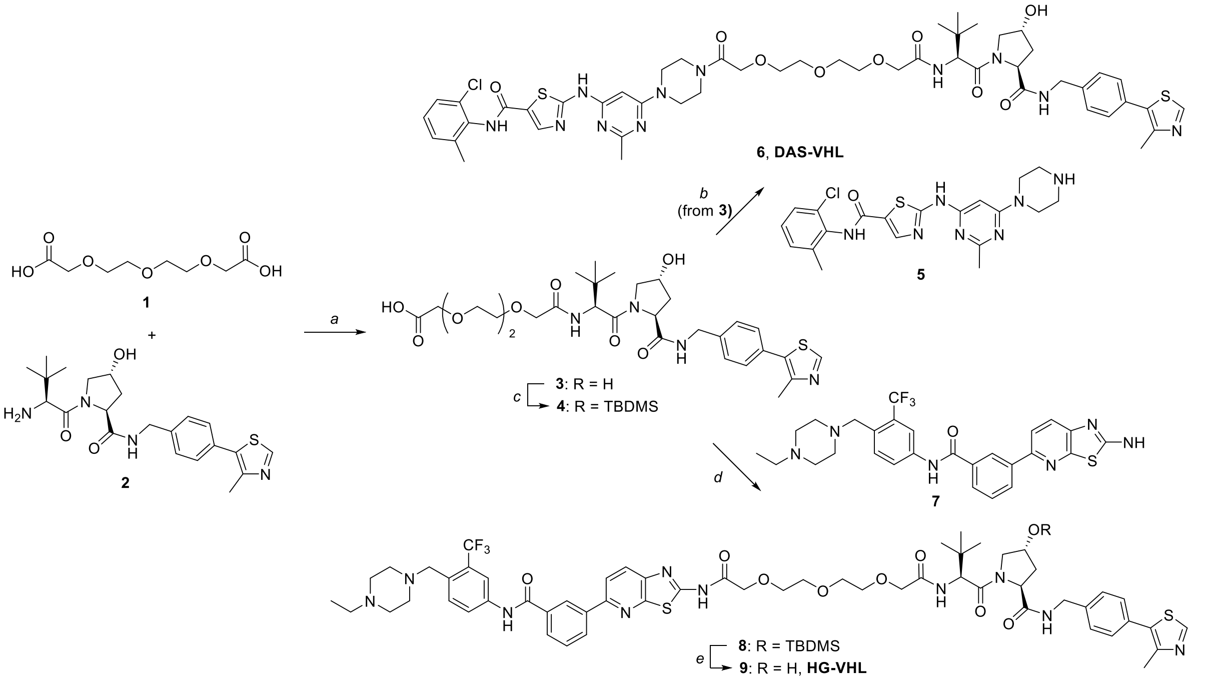

[a]Reagents and conditions: (a) EDC, HOBt, THF, DMF, rt, 86%; (b) **5**, HATU, DIPEA, MeCN, rt, 35%; (c) TBDMSCl, imidazole, THF, rt, 73%; (d) **7**, MNBA, Et_3N, DMAP, DMF, 0 °C, 59%; (e) TBAF, THF, rt, 58%.

Scheme S2. Synthesis of DAS-CRBN and HG-CRBN[a]

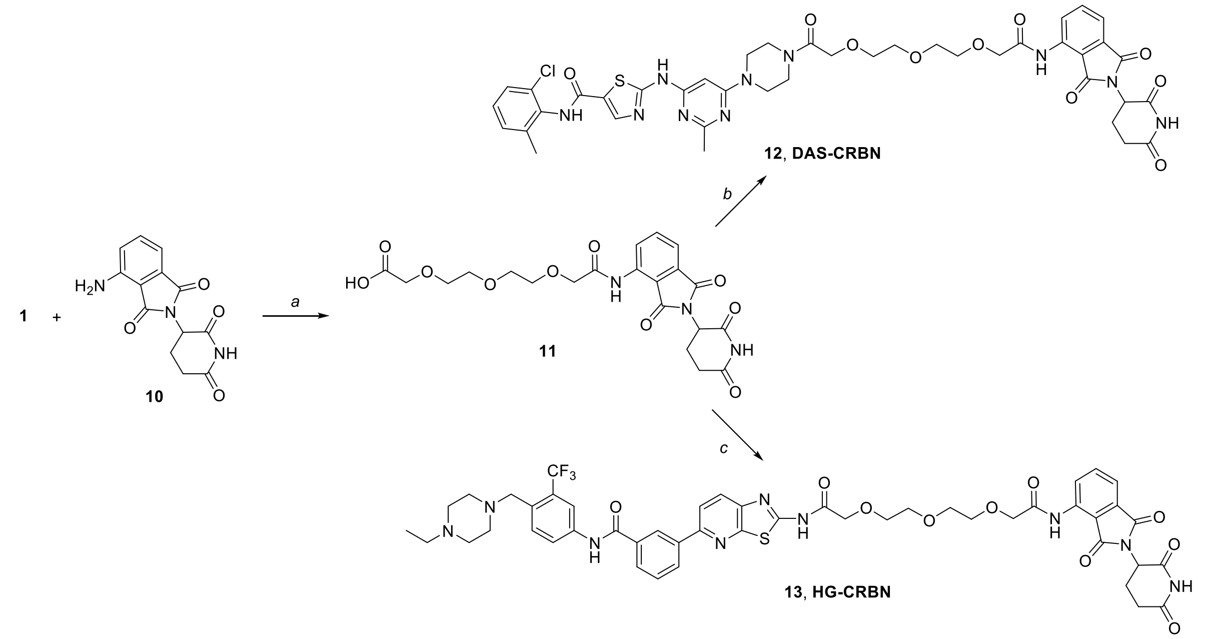

[a]Reagents and conditions: (a) **1**, $(COCl)_2$, DMF, THF rt; then **10**, THF, 60 °C, 91%; (b) **5**, HATU, DIPEA, DMF, rt, 31%; (c) **7**, HATU, DIPEA, DMF, rt, 14%.

Scheme S3. Synthesis of DAS-meIAP[a]

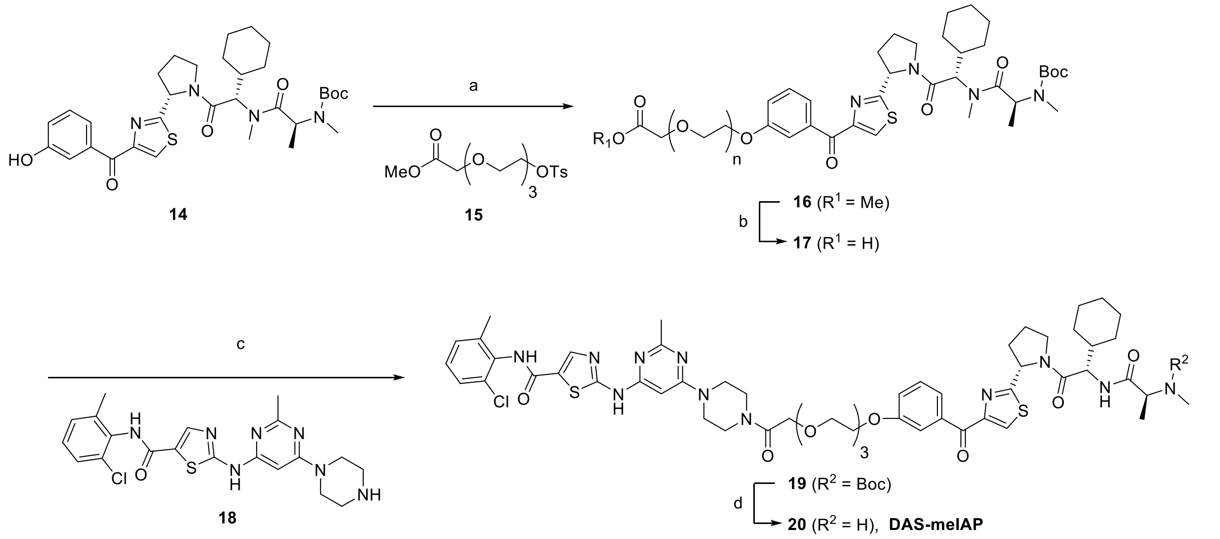

[a]Reagents and conditions: (a) **15**, K_2CO_3, DMF, 50 °C, 27%; (b) 4M LiOH aq., MeOH–THF–water, rt, quant.100%; (c) **18**, EDC, HOBt, DMF, rt, 93%; (d) TFA, rt, 80%.

Figure S1 Growth inhibitory effect of DAS-IAP, DAS-meIAP, and dasatinib in BCR-ABL nagative cells

a

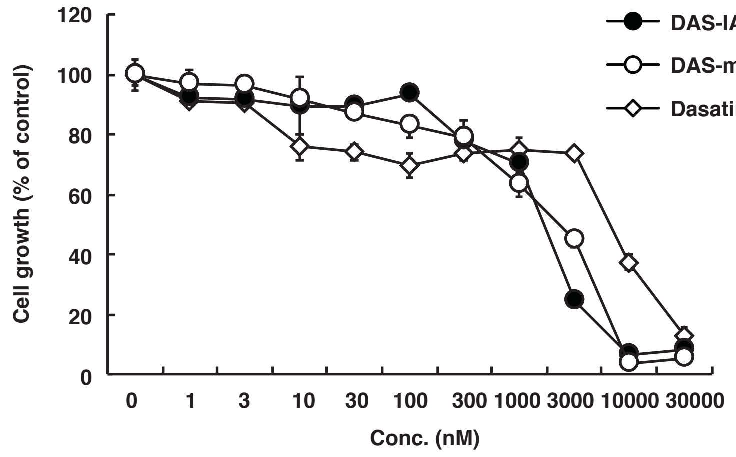

	Cell growth inhibition IC_{50} (μM)
DAS-IAP	1.72 ± 0.0649
DAS-meIAP	1.90 ± 0.108
Dasatinib	6.97 ± 0.458

b

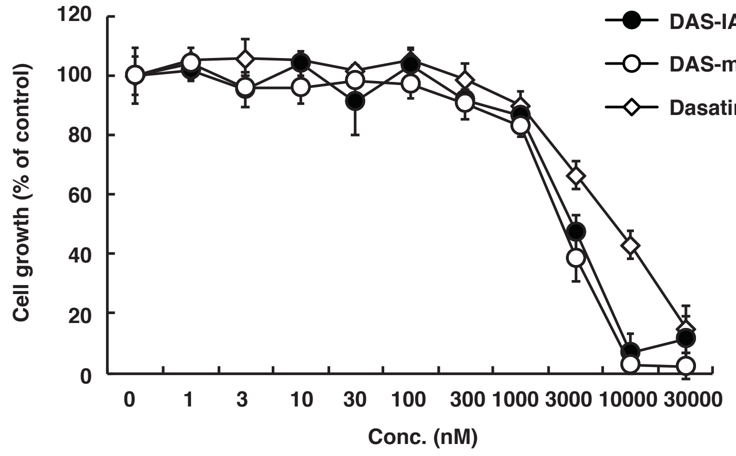

	Cell growth inhibition IC_{50} (μM)
DAS-IAP	2.87 ± 0.293
DAS-meIAP	2.44 ± 0.310
Dasatinib	6.79 ± 1.49

MOLT4 cells (a) and Jurkat cells (b) were incubated with the indicated concentration of the drugs for 48 h and subjected to the WST assay. IC_{50} values are presented as means ± SD (*n* = 3).

Figure S2 Original images of western blot analysis

Figure 1b

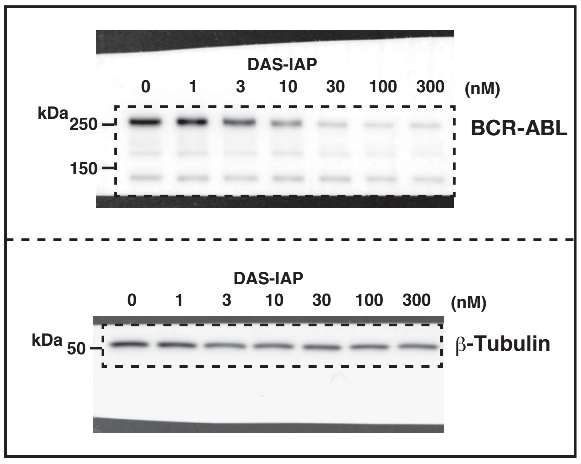

Figure 2b

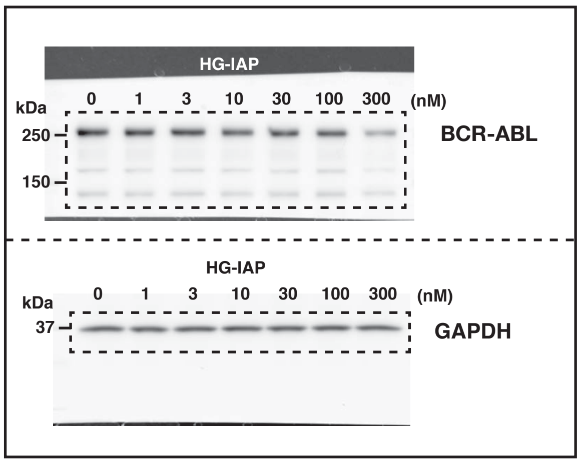

Figure 1c

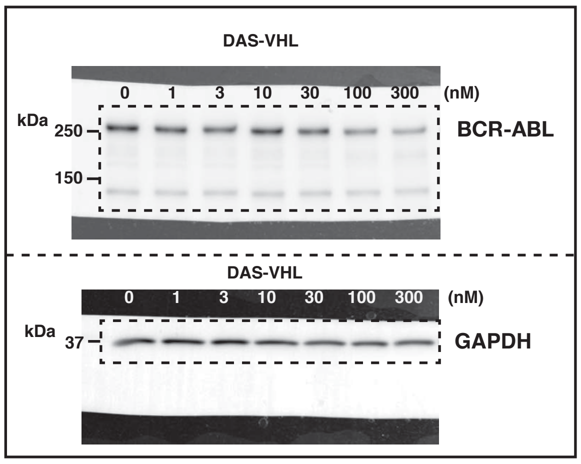

Figure 2c

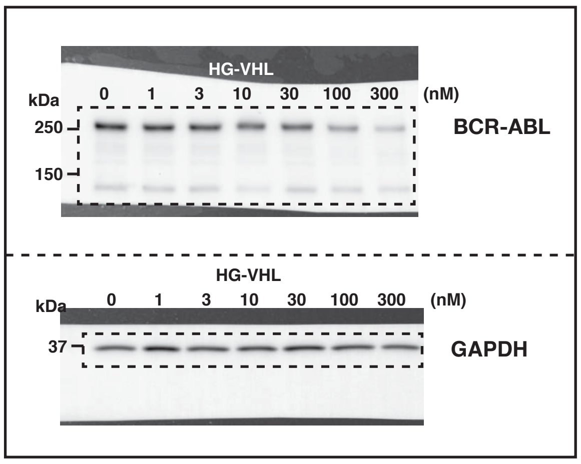

Figure 1d

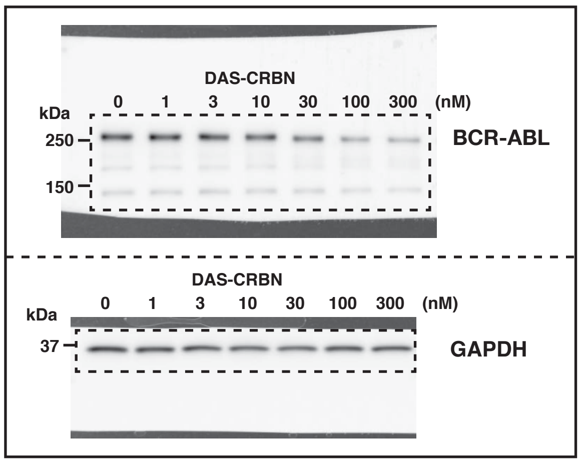

Figure 2d

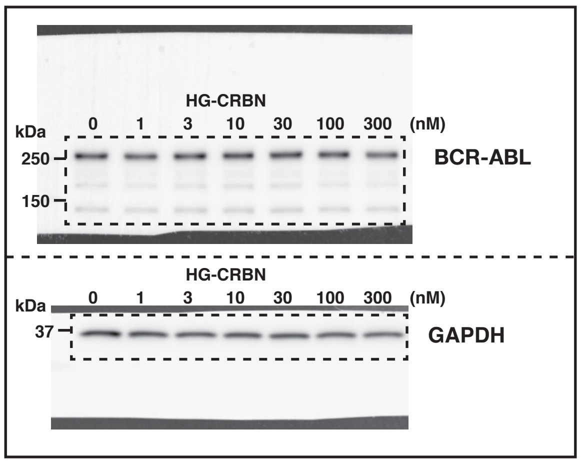

Figure S2 (continued)

Figure 3b

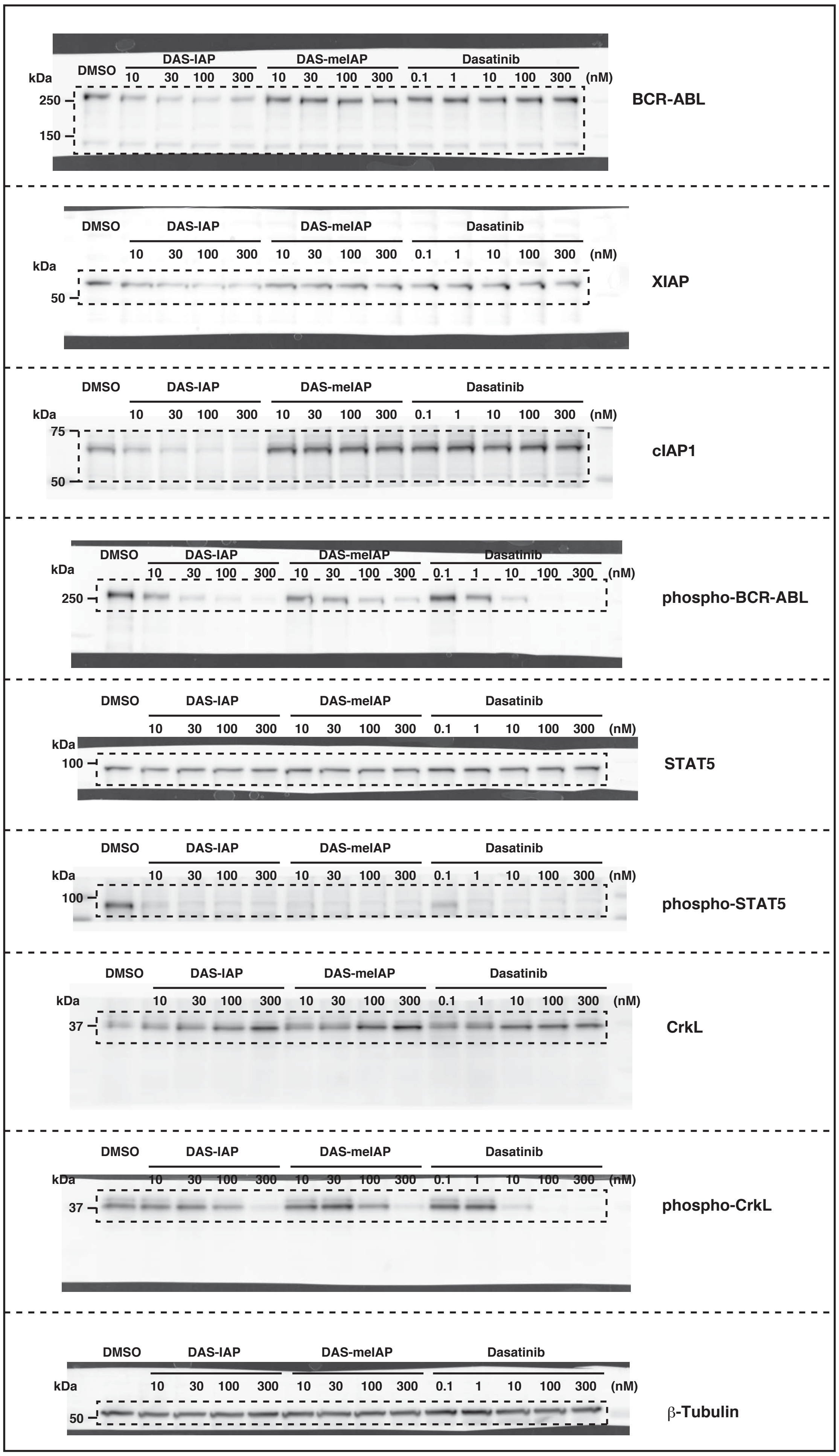

Figure S2 (continued)

Figure 4c

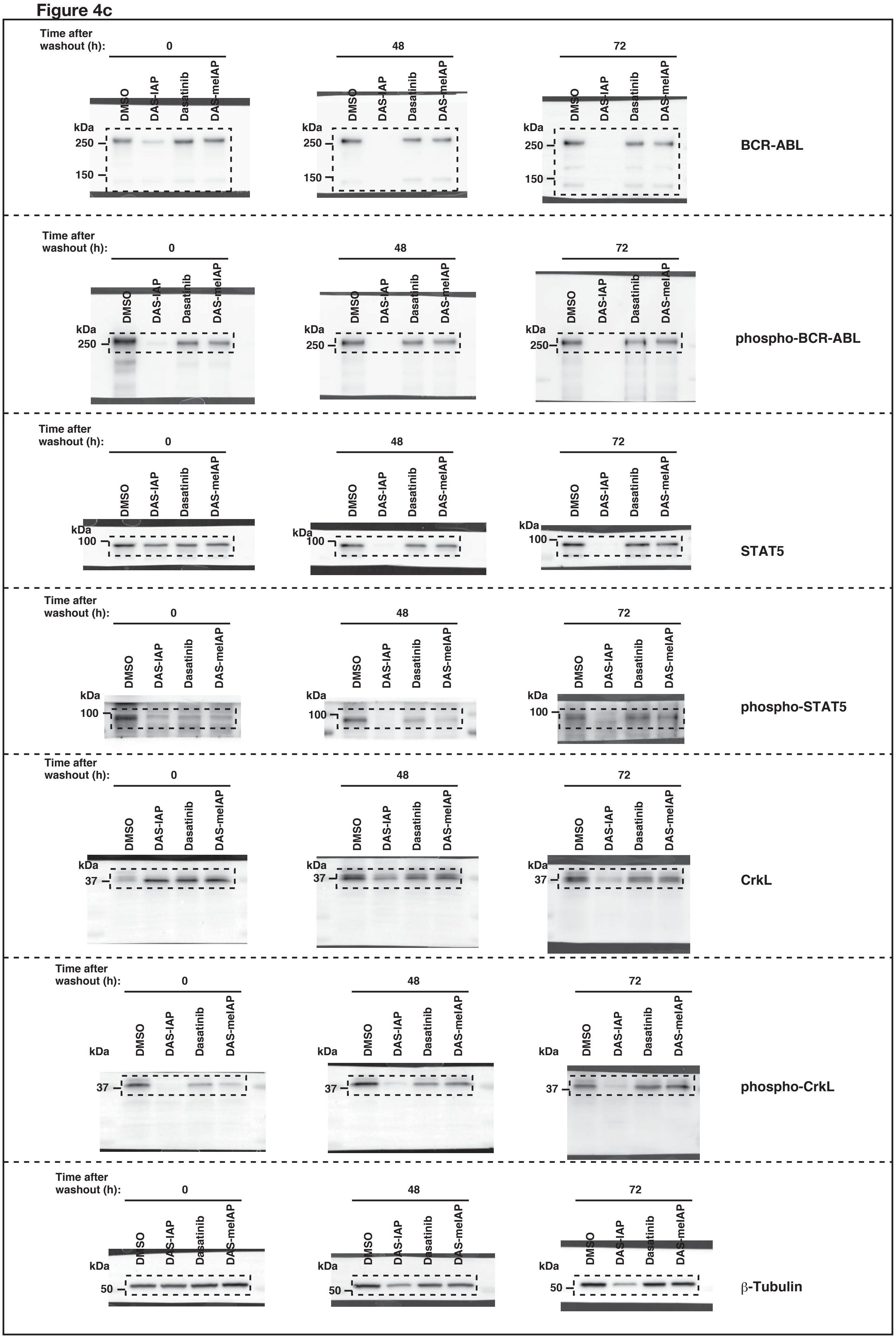

Figure S2 (continued)

Figure 5c

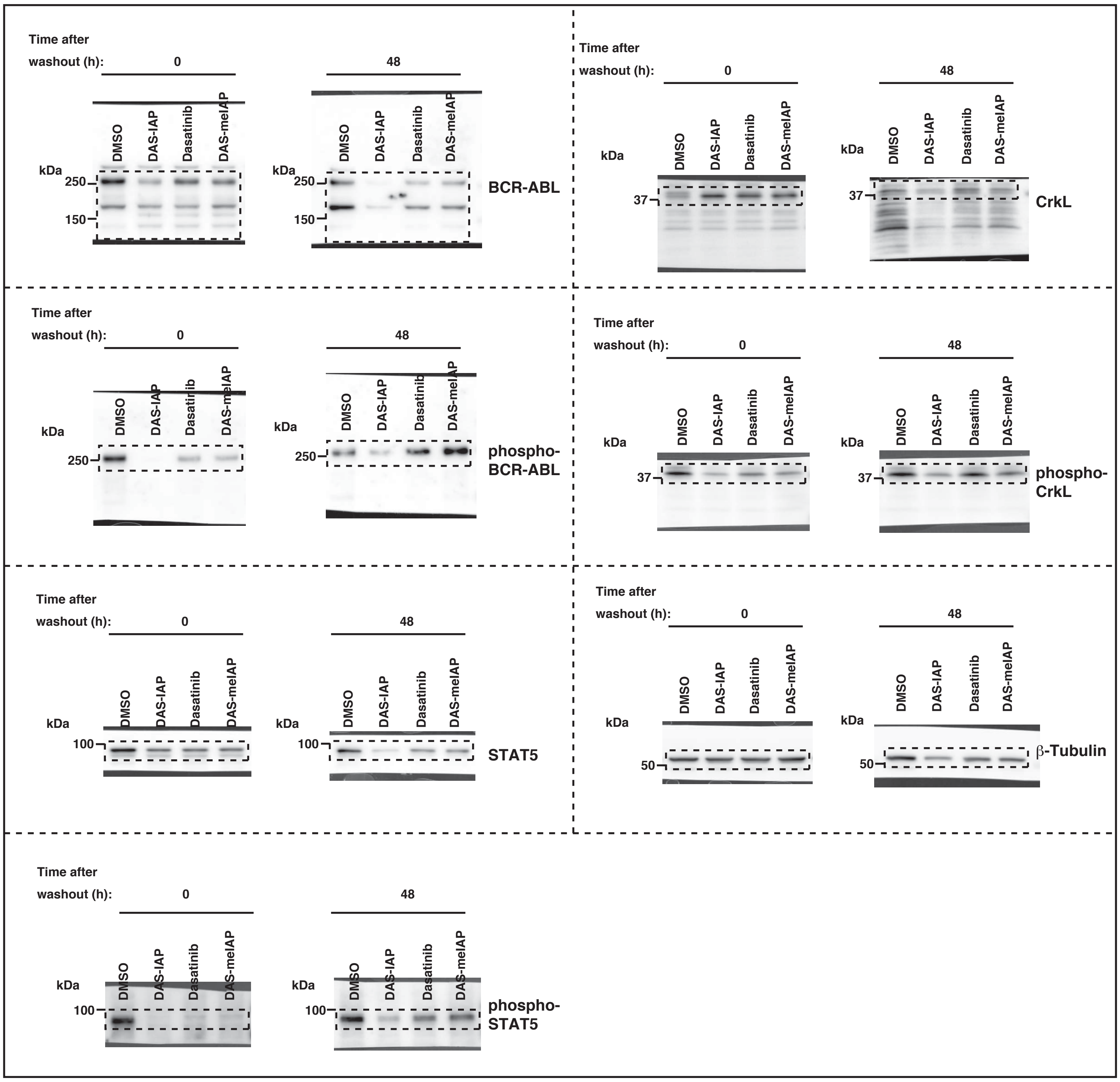

Figure S2 (continued)

Figure 6a

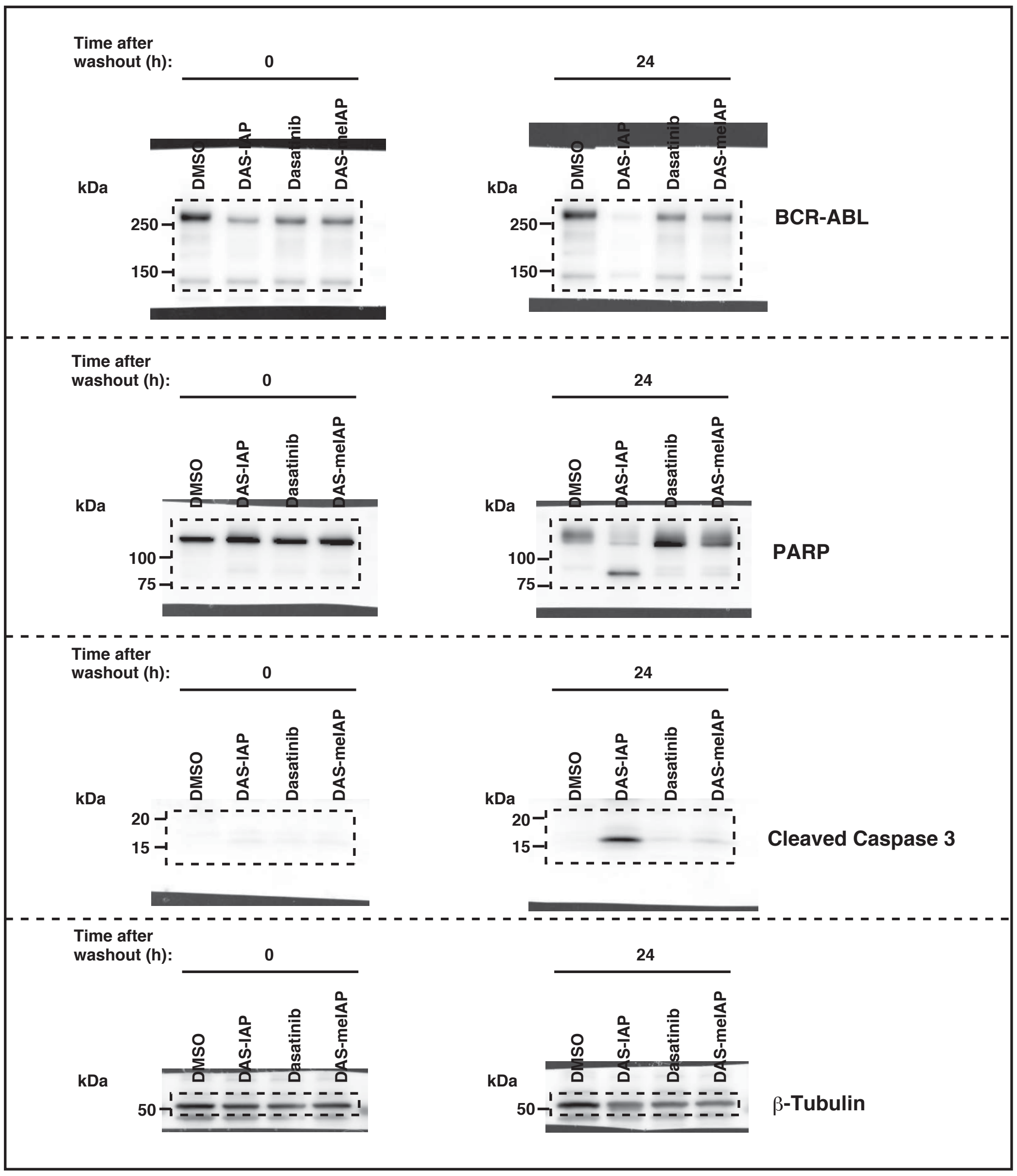

Figure 6b

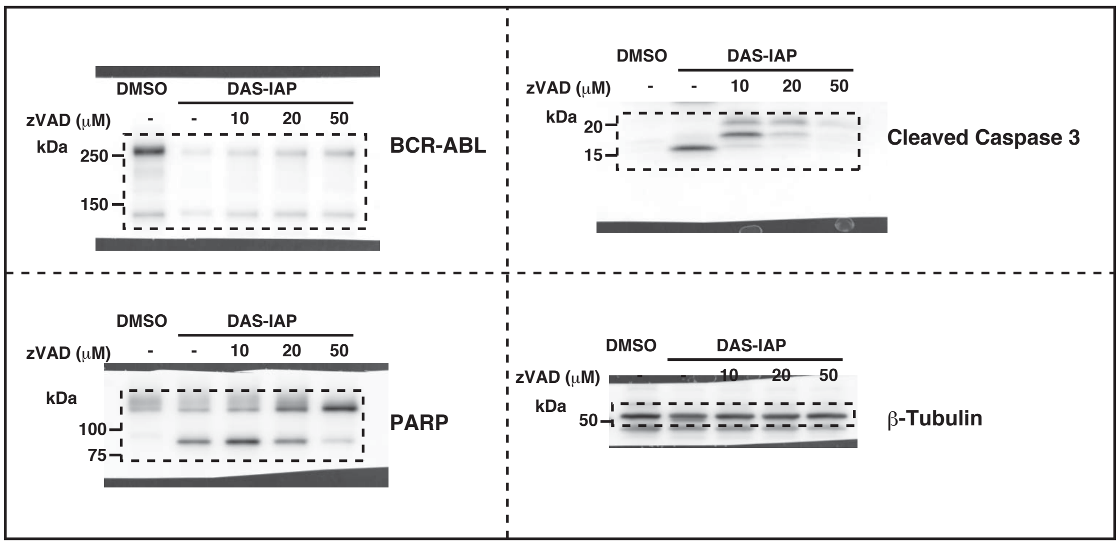

Supplement: Supplementary file 1 — Supplementary information [file 41598_2018_31913_MOESM1_ESM.pdf]
